# Development of Thalamo‐Telencephalic Circuits in a Reptile

**DOI:** 10.1002/cne.70203

**Published:** 2026-09-27

**Authors:** Michael B. Pritz

**Affiliations:** ^1^ Department of Biomedical Engineering University of Utah Salt Lake City Utah USA; ^2^ DENLABS Draper Utah USA

**Keywords:** *Alligator mississippiensis*, basal nuclei efferents, circuit evolution, lateral forebrain bundle, medial forebrain bundle, SV2 immunohistochemistry (AB_2315387)

## Abstract

Neuronal circuits are a core component required for proper brain function and organization. How these circuits are built remains incompletely understood. The vast majority of studies on forebrain development have investigated mammals. Whether the described observations found in mammals apply to other vertebrates is unknown. The present report examined the development of forebrain circuits that interconnect the dorsal thalamus and the telencephalon in *Alligator mississippiensis*. A tracer, neurobiotin, which is transported in both an anterograde and retrograde fashion, was used. Four circuits were identified after neurobiotin placement into either the diencephalon or in the forebrain bundles themselves. Three circuits ascended from the dorsal thalamus to the telencephalon. These were: the ipsilateral lateral forebrain bundle and the ipsilateral and contralateral medial forebrain bundles. The other circuit descended from the basal nuclei to connect with the dorsal thalamus. The first circuit to develop was the basal nuclei to dorsal thalamus circuit followed by the lateral forebrain bundle and lastly by the medial forebrain bundle. Because it preceded the formation of the three other circuits, the basal nuclei to the dorsal thalamus tract is suggested to serve as a scaffold for the other three circuits to follow. The formation of synapses by lateral forebrain bundle axons in the telencephalon was determined by immunohistochemistry using a synaptic vesicle glycoprotein. Synapse formation in the telencephalon from lateral forebrain bundle axons occurred before hatching. This suggested that information carried by the lateral forebrain bundle was critical for survival upon hatching. Medial forebrain bundle axon connections may become functional only after hatching.

## Graphical abstract

The development of forebrain connections was investigated in the *Alligator*. Four circuits were identified: ipsilateral basal nuclei to the dorsal thalamus, ipsilateral lateral forebrain bundle, and ipsilateral and contralateral medial forebrain bundle.

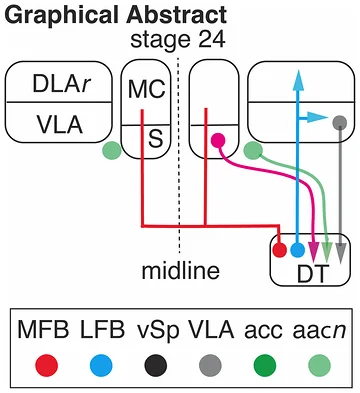

## 1 Introduction

The formation of the forebrain in amniotes (reptiles, birds, and mammals) is highly dependent on its connections. Of these, arguably the most consequential are the circuits between the dorsal thalamus and the telencephalon. The dorsal thalamus transfers nearly all messages from elsewhere in the brain to the telencephalon. The telencephalon then sends information back to the thalamus and to other brain centers (Guillery 2017).

Together, these thalamo‐telencephalic interconnections significantly influence an animal's behavior (Sherman and Guillery 2013).

While amniotes share several features in common, at least two significant differences in the organization of thalamo‐telencephalic interconnections differentiate mammals from sauropsids (reptiles and birds). These are: with the exception of turtles, the lack of reciprocal circuits in sauropsids, and the bilateral thalamo‐medial cortex circuit in sauropsids that is absent in mammals.

In mammals, an ipsilateral circuit using the internal capsule interconnects cortex and dorsal thalamus. In mammals, this thalamo‐cortical circuit is reciprocal; that is, a given dorsal thalamic nucleus projects to a specific cortical area. This particular cortical region then sends axons back to that same ipsilateral dorsal thalamic nucleus (Jones 2007).

Among sauropsids, only turtles possess this type of reciprocal circuit described in mammals. For the other reptiles, this feedback circuit originates in the basal nuclei, comparable, in part, to the striatum and globus pallidus of mammals (Parent 1976; Brauth and Kitt 1980; Belekhova et al. 1985; Desan 1988; Siemen and Künzle 1994; Perez‐Santana et al. 1997; Pritz 2024).

In turtles, a reciprocal, ipsilateral circuit between the dorsal geniculate nucleus and the pallium has been demonstrated. This circuit uses the lateral forebrain bundle (internal capsule of mammals) in both directions (W. C. Hall and Ebner 1970; J. A. Hall et al. 1977; Ouimet et al. 1985; Ulinski 1986; Heller and Ulinski 1987; Desan 1988; Mulligan and Ulinski 1990; Zhu et al. 2005). While a “reciprocal” thalamo‐pallial circuit has been described in birds, this pathway in birds differs from those found in turtles and mammals in two ways. One is that the projection from the thalamus to the pallium in birds is bilateral with both circuits using the lateral forebrain bundle (Karten et al. 1973). The other is that the ipsilateral circuit from pallium to the dorsal thalamus utilizes an entirely different circuit than does the ascending circuit (Karten et al. 1973; Reiner and Karten 1983).

The second difference in thalamo‐telencephalic connections between mammals and sauropsids is a bilateral projection from the dorsal thalamus to the medial cortex in sauropsids. This fiber path in sauropsids uses the medial forebrain bundle (Lohman and van Woerden‐Verkley 1978; Martinez‐Garcia and Lorente 1990; Desfilis et al. 2002) and is absent in mammals. However, this connection in sauropsids is not reciprocal; that is, no projection from medial cortex back to the dorsal thalamic nucleus of origin has been documented. Bilateral connections between the dorsal thalamus and cortex have been described in several mammals although details are incomplete.

Nevertheless, this circuit in mammals involves mostly medially located cortical structures and likely uses the internal capsule in both directions (Regidor and Divac 1992; Dinopoulos 1994; Künzle 1995; Goldman 1979; Beckstead 1979; Molinari et al. 1985; Preuss and Goldman‐Rakic 1987; Dermon and Barbas 1994; Carretta et al. 1996; Mathiasen et al. 2017).

An explanation for these differences between forebrain circuits among amniotes remains incompletely understood. One approach to investigate these variations in connections is to examine the development of these circuits. In mammals, the development of thalamo‐cortical interconnections has been the subject of innumerable studies. However, comparable analyses in sauropsids are few. In birds, with the exception of one study investigating the thalamo‐hyperstriatal system (Wu and Karten 1998), reports have focused on either a single circuit (Wu et al. 2000; Reyes‐Pinto et al. 2024) or on the overall pattern of dorsal thalamic projections to the telencephalon (Bielle et al. 2011; Tosa et al. 2015). In reptiles, studies have mainly examined fiber projections from nuclei that utilize the lateral forebrain bundle (Cordery and Molnàr 1999; Bielle et al. 2011; Tosa et al. 2015).

The development of two of the circuits that interconnect the thalamus with the telencephalon has received little attention in sauropsids. These are the development of the bilateral medial forebrain bundle circuit and the development of the circuit between the basal nuclei and the dorsal thalamus.

The present study fills these gaps in knowledge by investigating the development of the following four circuits that interconnect the dorsal thalamus with the telencephalon.

These are: (1) the ipsilateral connection from the dorsal thalamus to the telencephalon; (2) the ipsilateral and (3) contralateral circuit from the dorsal thalamus to the medial cortex; and (4) the circuit from the basal nuclei to the diencephalon. *Alligator mississippiensis* was chosen as a representative reptile for two reasons. One is because *Alligator* is a member of the reptilian group most closely related to birds (Whetstone and Martin 1979; Hedges 1994) where considerable developmental experiments have been performed, and their results can be used for comparison. The other is because the time course for differentiation of the dorsal thalamus (Pritz 2025a) and the telencephalon is known (Pritz 2025b, 2025c).

This study addresses the following questions. First, what is the time course for the development of each of these four circuits? Second, is the development of the medial and lateral forebrain bundles similar or different? Third, how does the time course of development of the circuits between the basal nuclei and the dorsal thalamus compare with development of projections from the dorsal thalamus to the telencephalon? Fourth, how do these results in *Alligator* compare with similar studies in other amniotes?

## 2 Methods and Materials

The experiments in this report were approved by the animal use committee of Indiana University (Protocol Approval 10516). This protocol followed the guidelines of the National Institutes of Health.

### 2.1 Animals

*Alligator* eggs were obtained as a clutch from the Rockefeller Wildlife Refuge at Grand Chenier, Louisiana. Upon arrival at the laboratory, eggs were incubated (Model 1535, VWR Scientific, Philadelphia, PA) at 30°C and housed in a 2:1 mixture of vermiculite and water.

### 2.2 Embryo Harvesting

Embryos were sacrificed at pre‐arranged times based on approximate developmental stages for *Alligator mississippiensis* (Ferguson 1985). Processing was as follows. First, eggs were candled to determine viability. Viable embryos were then freed from surrounding tissues using microsurgical techniques under a dissecting microscope (Zeiss, Germany) and staged (Ferguson 1985). Embryos were euthanized by transection at the cervico‐medullary junction. Embryos used for tract tracing experiments were injected with tracer immediately, whereas those studied for immunohistochemistry were placed in Dent's solution (80% methanol; 20% dimethylsulfoxide) and stored at −80°C.

### 2.3 Tract Tracing Experiments

The apparatus and techniques for tract tracing were similar to those used in a previous study (Pritz 2026). This methodology is referred to as ex vivo tract tracing. Details are listed below.

Neurobiotin (Vector Laboratories, Newark, CA) applications were of two types: coated beads or crystals. Coated beads were made by adding ∼1 µL of 0.1 M phosphate‐buffered saline (PBS) at pH 7.2 to crystals of neurobiotin on a microscope slide.

Sephadex beads (100–300 µm in diameter; Sigma, St. Louis, MO) were then placed into this neurobiotin slurry, separated, and then air‐dried. After adequate drying, coated beads were attached to an insect pin coated with ficoll (Davis and Agranoff 1977) and stored at 2°C–4°C until used. Neurobiotin crystals were attached to a ficoll‐coated insect pin and stored at 2°C–4°C until used.

After tracer injection, the brain was placed into a device for ex vivo transport of tracer. A photograph and description of this experimental chamber is available elsewhere (Figure 1, Pritz 2026). The chamber was filled with reptilian cerebrospinal that covered the injected brains and was changed daily. The tissue was continuously bathed in physiological saline containing (in mM): 100 NaCl, 6 KCl, 40 NaHCO_3_, 2.6 CaCl_2_, 1.6 Mg Cl_2_, and 20 glucose. A combination of 95% oxygen/5% carbon dioxide continuously aerated the chamber, which was kept at 0°C–6°C. The time in the chamber varied between 24 and 100 h (Table 1).

**FIGURE 1:**
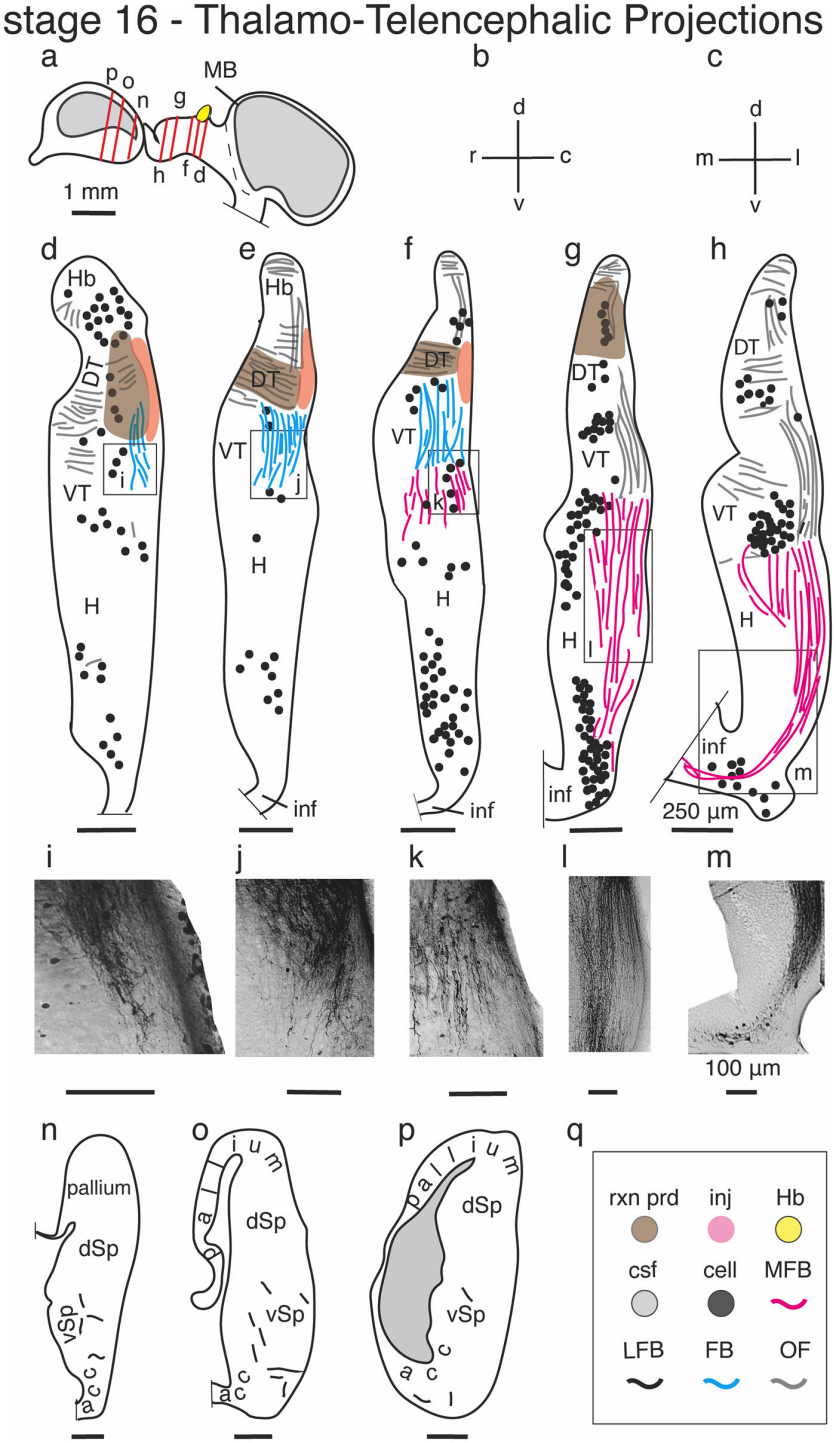
Stage 16 thalamo‐telencephalic projections. Fiber tract labeling after a tracer injection into the diencephalon is illustrated (d–h). The injection site (d–f) and brown reaction product (d–g) are shown. The approximate location and plane of section for these transverse sections are indicated on a schematic parasagittal outline of a Stage 16 embryo (a). Photographs of forebrain tracts (i–m) are from the enclosed boxes (d–h). Bar scale for each line drawing is the same and is indicated (h). Bar scale for each photo is the same and is shown (m). The orientation for the sagittal schematic (a) is indicated (b). The orientation for the transverse sections is shown (c). Coding scheme for the transverse section line drawings is indicated (q). acc, accumbens area; c, caudal; csf, cerebrospinal fluid; d, dorsal; D, diencephalon; DT, dorsal thalamus; FB, forebrain bundles; H, hypothalamus; Hb, habenula; inf, infundibulum; inj, injection site; l, lateral; LFB, lateral forebrain bundle; m, medial; MB, midbrain; MFB, medial forebrain bundle; OF, other fibers; r, rostral; rxn prd, brown reaction product; v, ventral; VT, ventral thalamus (prethalamus).

**TABLE 1:** Neurobiotin tract tracing experiments.

Stage	Injection	Survival	4%PF	Fix*	PBS*	Block/Fix*	Plane	Thickness	Injection	Comment
16	Crystal	50 h	79 h	39 h	8 h	64 h	T	60 µm	Figures 1, 2, and 12	
16	Crystal	49 h	79 h	39 h	8 h	64 h	S	60 µm	Figure 9b–e	
16	Crystal	47 h	49 h	—	5 h	120 h	S	60 µm	D+Teg	Local
18	Crystal	46 h	52 h	64 h	7 h	87 h	T	60 µm	Figures 3 and 13	
18	Bead	46 h	52 h	64 h	7 h	89 h	S	60 µm	Figure 9g,h	
18	Crystal	52 h	136 h	—	7 h	120 h	S	60 µm	DT	LFB+Retro. cells
19.5	Crystal	71 h	138 h	—	7 h	96 h	S	60 µm	DT	LFB+Retro. cells
19.5	Crystal	71 h	139 h	—	7 h	144 h	T	100 µm	DT/VT	Local
19.5	Crystal	76 h	48 h	48 h	4 h	96 h	S	60 µm	Figure 9j–m	
20	Bead	70 h	80 h	39 h	6 h	72 h	T	60 µm	FB+VT	LFB+Retro. cells
20	Crystal	69 h	79 h	39 h	6 h	72 h	T	60 µm	Figures 4 and 14	
20	Bead	69 h	79 h	39 h	6 h	72 h	S	60 µm	DT+II Tr	PT+TeO cells+fibers
21.5	Crystal	76 h	119 h	43 h	8 h	72 h	T	60 µm	DT+PT	Local
21.5	Crystal	75 h	119 h	43 h	8 h	168 h	T	100 µm	D	Local
21.5	Crystal	75 h	119 h	43 h	8 h	312 h	S	100 µm	D	No transport
22.5	Crystal	49 h	—	163 h	—	192 h	S	100 µm	D	Local
22	Crystal	76 h	48 h	48 h	4 h	96 h	T	60 µm	Figures 5, 6, and 15	
22.5	Crystal	75 h	48 h	48 h	4 h	96 h	S	60 µm	Figure 10b–d	
22.5	Bead	74 h	48 h	48 h	4 h	96 h	S	60 µm	DT	LFB+Retro.cells
22.5	Crystal	87	217 h	—	6 h	168 h	S	100 µm	D	Retro. cells
24	Crystal	73 h	77 h	99 h	7 h	96 h	T	60 µm	D+II Tr	LFB+Retro.cells
24	Bead	72 h	77 h	99 h	7 h	96 h	S	60 µm	Figure 10f,g	
24	Bead	72 h	77 h	99 h	7 h	96 h	S	60 µm	DT	LFB+Retro. cells +
24	Crystal	100 h	379 h	—	—	168 h	S	100 µm	D	Retro. cells
24	Crystal	24 h	120 h	—	—	72 h	T	50 µm	Figures 7 and 16	
24	Crystal	27 h	120 h	—	—	72 h	T	50 µm	D	LFB+Retro.cells
24	Crystal	100 h	379 h	—	—	168 h	T	100 µm	Hb+SM	Local+Retro. cells
24	Crystal	99 h	379 h	—	—	168 h	T	100 µm	D	Local
26–28	Crystal	74 h	241 h	42 h	6 h	240 h	T	100 µm	DT	Local
26–28	Crystal	73 h	241 h	42 h	6 h	240 h	T	100 µm	DT	Local
26–28	Crystal	74 h	241 h	42 h	6 h	288 h	S	100 µm	DT	Local
26–28	Crystal	89 h	120 h	69 h	6 h	90 h	T	80 µm	D	Local +VLA
26–28	Crystal	45 h	1559 h	21 h	7 h	90 h	T	80 µm	MB Teg	Local +Retro. cells
26–28	Crystal	45 h	2044 h	94 h	3 h	19 h	T	80 µm	MB Teg	Local +Retro. cells
26–28	Crystal	73 h	72 h	72 h	5 h	48 h	T	60 µm	Figures 8 and 17	
26–28	Crystal	73 h	72 h	72 h	5 h	48 h	S	60 µm	Figure 10i–l	
26–28	Crystal	72 h	24 h	72 h	5 h	48 h	S	60 µm	DT	LFB+Retro.cells
26–28	Crystal	47 h	864 h	44 h	4 h	96 h	T	80 µm	PT	Local+VLA+Teg
26–28	Crystal	46 h	1224 h	50 h	3 h	48 h	T	80 µm	PT	Local +VLA

*Note:* D, diencephalon; DT, dorsal thalamus; Fix*, fixative‐ 4%paraformaldehyde (PF)/30% sucrose/0.1 M phosphate‐buffered saline at pH 7.2 (PBS); h, hours; Hb, habenula; Local, local transport only; LFB, labeling of ascending lateral forebrain fibers; PT, pretectum; MB, midbrain; Retro. cells, retrogradely labeled telencephalon cells; S, sagittal; SM, stria medullaris; T, transverse; Teg, tegmentum; TeO, optic tectum; VLA, ventrolateral area; VT, ventral thalamus; II Tr, optic tract; —, no data or not done.

At the termination of the ex vivo experiment, brains were placed in 4% paraformaldehyde (PF)/PBS before transfer to 4% PF/30%sucrose/PBS (fixative).

Brains were then immersed in PBS before embedding in gelatin (7.5% gelatin in 15% sucrose/distilled water). Times in different processing steps varied based on availability of technicians to process the tissue (Table 1). Gelatin‐embedded blocks were placed into fixative. Brains were sectioned on a sliding microtome (AO Optical, Buffalo, NY) either transversely or sagittally. Sections were placed in PBS if processed immediately or stored in 4%PF/PBS at 2°C–4°C if processed later. Free‐floating sections were thoroughly washed in PBS before beginning the protocol, between all steps, and after the last step. Sections were processed in the following sequence: (1) 3% hydrogen peroxide/PBS for 10 min; (2) 0.5% Triton‐X‐100(TX)/PBS for 30 min; and (3) avidin‐biotin complex (Vector) at a concentration of 1:100/0.5%TX/PBS overnight. The reaction product was visualized by section immersion in a solution composed of 15 mg diaminobenzidine/30 mL PBS to which 10 µL of 30% hydrogen peroxide was added for 5 min. Sections were then mounted on chrome alum‐coated slides, air‐dried, dehydrated through a series of graded alcohols, cleared in xylene, and cover‐slipped with Permount (Fisher Pittsburgh, PA). Information about individual experiments is detailed in Table 1.

### 2.4 SV2 Immunohistochemistry

Embryonic brains between stages 16 and 26–28 were examined to detect the expression of synaptic vesicle glycoprotein 2A (synaptic vesicles; SV2; AB_2315387; Developmental Studies Hybridoma Bank, University of Iowa, Iowa City, IA) raised in mouse (mouse SV2 antibody). This antibody recognized immunoblots of lizard brain extracts (Romero‐Alemán et al. 2010)

Brains were kept at −80°C. Stored brains were gradually warmed, placed in decreasing concentrations of methanol beginning at 100% methanol, and last into PBS. Brains were transferred to a solution of 30% sucrose/2%formalin/PBS at 2‐−4°C for 2 days prior to embedding in gelatin. Gelatin blocks were placed into 30% sucrose/2%formalin/PBS overnight before sectioning. Brains were then sectioned on a sliding microtome and placed into individual wells containing PBS. Free‐floating sections were processed sequentially in the following solutions after thorough washing in PBS: (1) 5% normal horse serum (NHS; Sigma, St. Louis, MO)/0.2% TX/PBS for 1 h; (2) mouse SV2 antibody in 5%/NHS/TX/PBS at a concentration of 1:1000 at room temperature for 1 h and then overnight at 2°C–4°C; (3) horse‐anti‐mouse IgG (Vector) at a concentration of 1:200 in 5%NHS/TX/PBS for 2 h; (4) avidin biotin complex (Vector) at a concentration of 1:100 for 2 h; (5) diaminobenzidine 15 mg/30 mL PBS to which 10 µL hydrogen peroxide was added for 5 min. Between each step and after the last step, sections were washed thoroughly in PBS. Sections were then mounted on slides, air‐dried, dehydrated through a series of graded alcohols, cleared in xylene, and cover‐slipped (Cytoseal 60, Fisher Scientific, Pittsburgh, PA).

Controls were the following. One was absent staining in the basal nuclei or their anlage and absent staining in the rostral dorsolateral area or its anlage at Stages 16–20, whereas SV2 (+) puncta were seen in the optic tectum and hypothalamus at Stage 20. The other was immunoreactive staining of fresh rat brain using the same protocol and same concentration of the mouse SV2 antibody used to stain the ventrolateral and rostral dorsolateral areas at Stages 22 to 26–28. Additional experimental details are listed in Table 2.

**TABLE 2:** SV2 experiments.

Stage	Thickness (µm)	Plane of section	Basal nuclei or anlagen staining (yes/no)	Number
16	40	Transverse	No	1
18	40	Transverse	No	1
20	40	Transverse	No	1
22	40	Transverse	Yes	1
24	40	Transverse	Yes	1
26–28	40	Transverse	Yes	1
26–28	30	Transverse	Yes	2

### 2.5 Morphological Analysis

Line drawings of selected sections were viewed under brightfield illumination using a Leitz Dialux microscope (Wetzlar, Germany) with a drawing tube attachment. Drawn sections were scanned and imported into Adobe Illustrator (2025, version 29.21) where they were traced and assembled into composite figures.

Labeled structures after diencephalon injections were best viewed at higher magnification, for example, x20, x40, with the aperture diaphragm closed nearly completely.

Fibers were extremely fine at all stages examined. At all stages, but particularly at early stages, Stages16–22, varicosities and swellings along fibers could be misconstrued as puncta at lower magnification because fine fibers between varicosities would not be visualized.

Photos of sections used a digital camera (Infinity 8–8 C Color 8 MP microscope camera, Teledyne Lumenera, Evident Scientific, Ottawa, Ontario, Canada) attached to an Olympus BH2 microscope (Olympus Corporation, Lake Success, NY). Images were imported into Adobe Illustrator where they were cropped and assembled into figures. Only brightness and contrast were changed. Similar to sections viewed for line drawings, identification of puncta and immunopositive SV2 elements could only be seen at higher magnification, for example, x20 and x40. In addition, nearly complete closing of the aperture iris diaphragm ring enabled better image resolution and photos.

## 3 Results

### 3.1 General Comments

Other structures besides the medial and lateral forebrain bundles and the basal nuclei were labeled by the tracer injections into the diencephalon. These other nuclei and tracts included other parts of the diencephalon, the hypothalamus, the preoptic area, and the infundibulum. While these retrogradely labeled cells and fiber bundles were shown in the accompanying figures (Figures 1, 2, 3, 4, 5, 6), these results were not discussed. Similarly, photographs of the lateral forebrain bundle (Figures 7 and 8) focused on its course and termination. The goal of this report was to determine the time course and the nature of the formation of thalamo‐telencephalic interconnections. These other tracts and brain areas were clearly important for understanding the totality of diencephalic and forebrain connections and organization. However, these additional observations were outside the focus of this report and were not discussed further.

**FIGURE 2:**
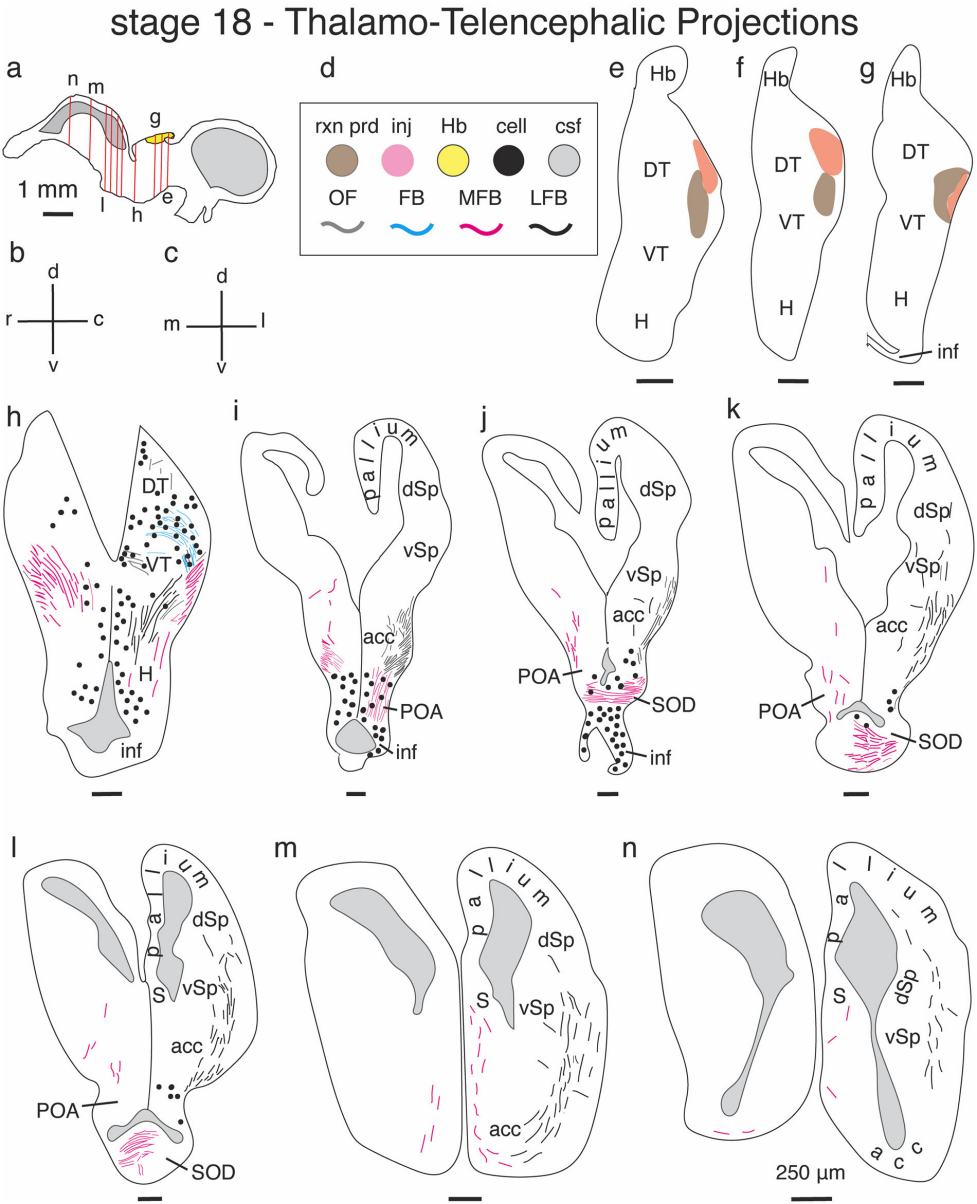
Stage 18 thalamo‐telencephalic projections. Fiber tract labeling after a tracer injection into the diencephalon is illustrated (h–n). The injection site and brown reaction product surround (e–g) are shown. The approximate location and plane of section for these transverse sections are indicated on a schematic parasagittal outline of a stage 18 embryo (a). Bar scale for each line drawing is the same and is indicated (n). The orientation for the sagittal schematic (a) is indicated (b). The orientation for the transverse sections is shown (c). Coding scheme for the transverse section line drawings is indicated (d). acc, accumbens area; c, caudal; csf, cerebrospinal fluid; d, dorsal; dSp, dorsal subpallium; DT, dorsal thalamus; H, hypothalamus; Hb, habenula; inf, infundibulum; inj, injection site; l, lateral; LFB, lateral forebrain bundle; m, medial; MFB, medial forebrain bundle; OF, other fibers; POA, preoptic area; r, rostral; rxn prd, brown reaction product; S, septum; SOD, supraoptic decussation; v, ventral; vSp. ventral subpallium; VT, ventral thalamus (prethalamus).

**FIGURE 3:**
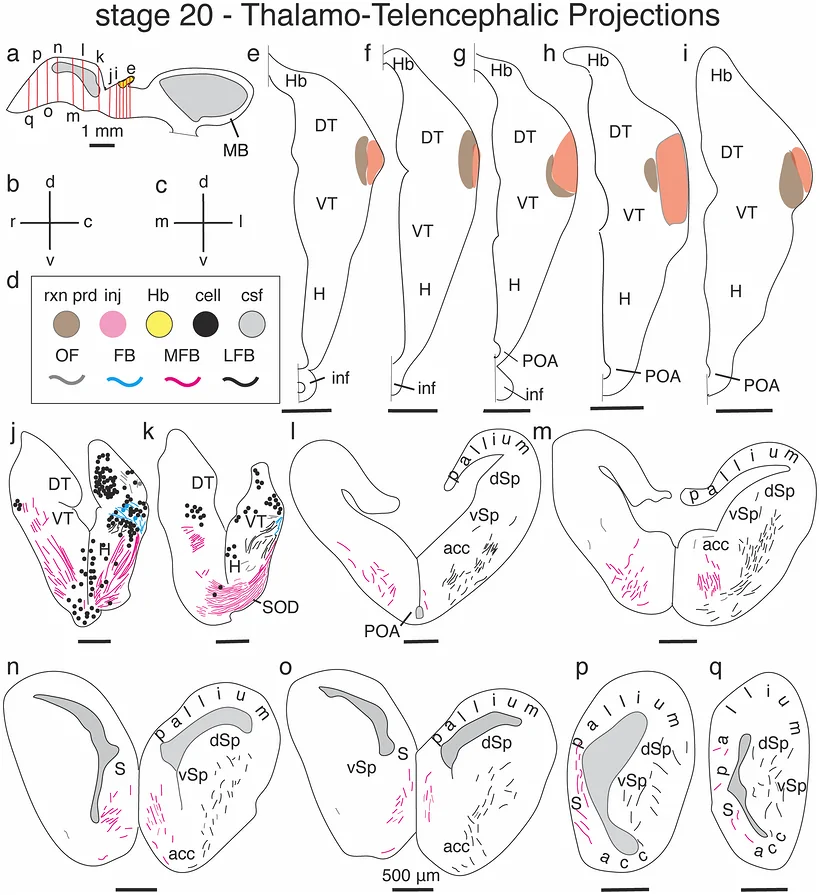
Stage 20 thalamo‐telencephalic projections. Fiber tract labeling after a tracer injection into the diencephalon is illustrated. Only the ipsilateral hemisphere was shown in the two most rostral sections (p, q) because no staining was seen contralaterally. The injection site and brown reaction product surround are shown (e–i). The approximate location and plane of section for these transverse sections (e–q) are indicated on a schematic parasagittal outline of a stage 20 embryo (a). Bar scale for each line drawing is the same and is indicated (o). The orientation for the sagittal schematic (a) is indicated (b). The orientation for the transverse sections is shown (c). Coding scheme for the transverse section line drawings is indicated (d). acc, accumbens area; c, caudal; csf, cerebrospinal fluid; d, dorsal; dSp, dorsal subpallium; DT, dorsal thalamus; H, hypothalamus; Hb, habenula; inf, infundibulum; inj, injection site; l, lateral; LFB, lateral forebrain bundle; m, medial; MB, midbrain; MFB, medial forebrain bundle; OF, other fibers; POA, preoptic area; r, rostral; rxn prd, brown reaction product; S, septum; SOD, supraoptic decussation; v, ventral; vSp. ventral subpallium; VT, ventral thalamus (prethalamus).

**FIGURE 4:**
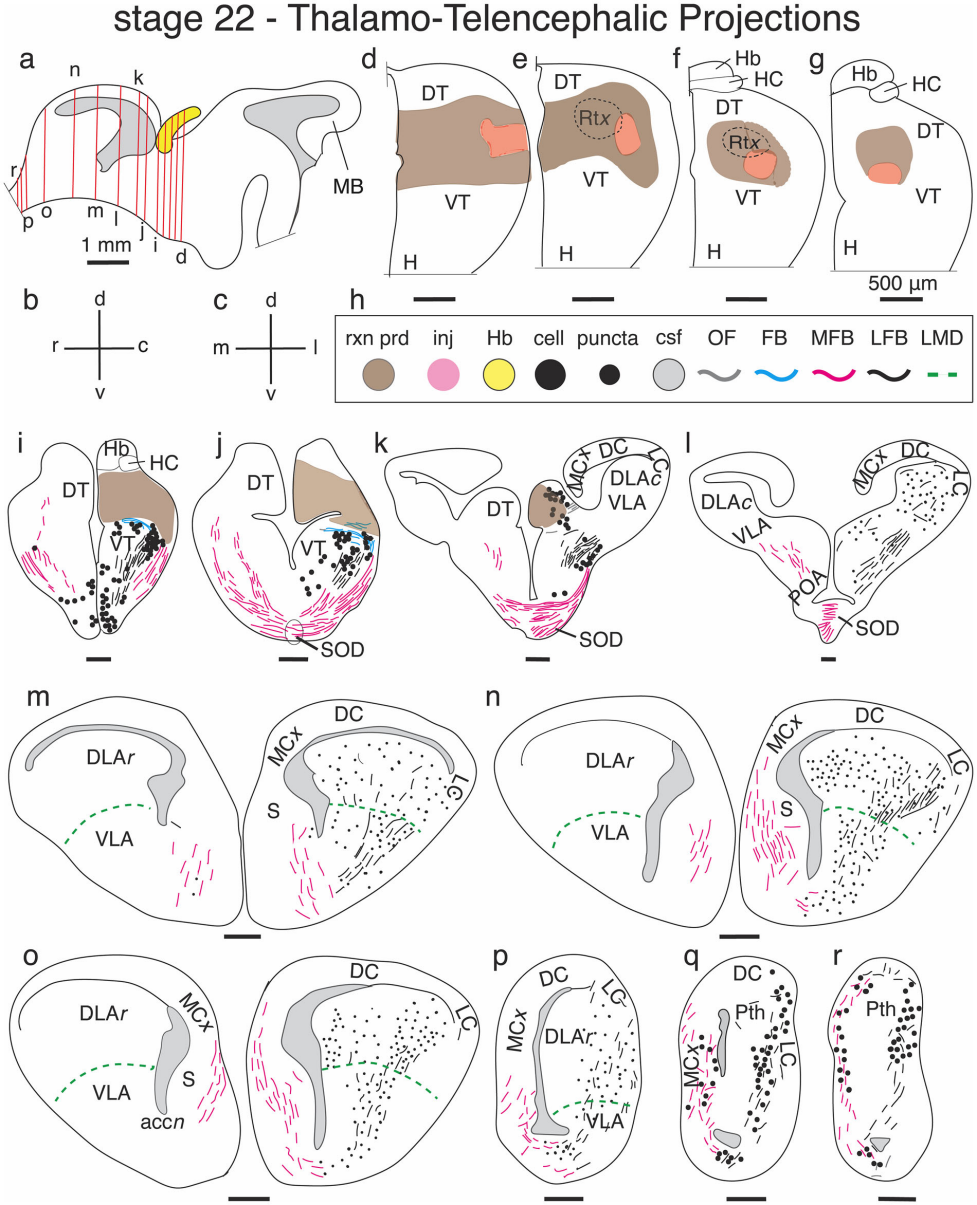
Stage 22 thalamo‐telencephalic projections. Fiber tract labeling after a tracer injection into the diencephalon is illustrated. Only the ipsilateral hemisphere was shown in the most rostral section (p–r) because no staining was seen contralaterally. The injection site (d–g) and brown reaction product (d–g, i, j) are shown. The approximate location and plane of section for these transverse sections (d–g, i–r) are indicated on a schematic parasagittal outline of a Stage 22 embryo (a). Bar scale for each line drawing is the same and is indicated (g). The orientation for the sagittal schematic (a) is indicated (b). The orientation for the transverse sections is shown (c). Coding scheme for the transverse section line drawings is indicated (h). acc*n*, accumbens nucleus; c, caudal; csf, cerebrospinal fluid; d, dorsal; DC, dorsal cortex; DLA*c*, caudal dorsolateral area; DLA*r*, rostral dorsolateral area; DT, dorsal thalamus; FB, forebrain bundle; H, hypothalamus; Hb, habenula; HC, habenular commissure; inf, infundibulum; inj, injection site; l, lateral; LC, lateral cortex; LFB, lateral forebrain bundle; LMD, dorsal medullary lamina; m, medial; MB, midbrain; MC*x*, medial cortex anlage; MFB, medial forebrain bundle; OF, other fibers; POA, preoptic area; r, rostral; Rt*x*, nucleus rotundus anlage; rxn prd, brown reaction product; S, septum; SOD, supraoptic decussation; v, ventral; VLA, ventrolateral area; VT, ventral thalamus (prethalamus).

**FIGURE 5:**
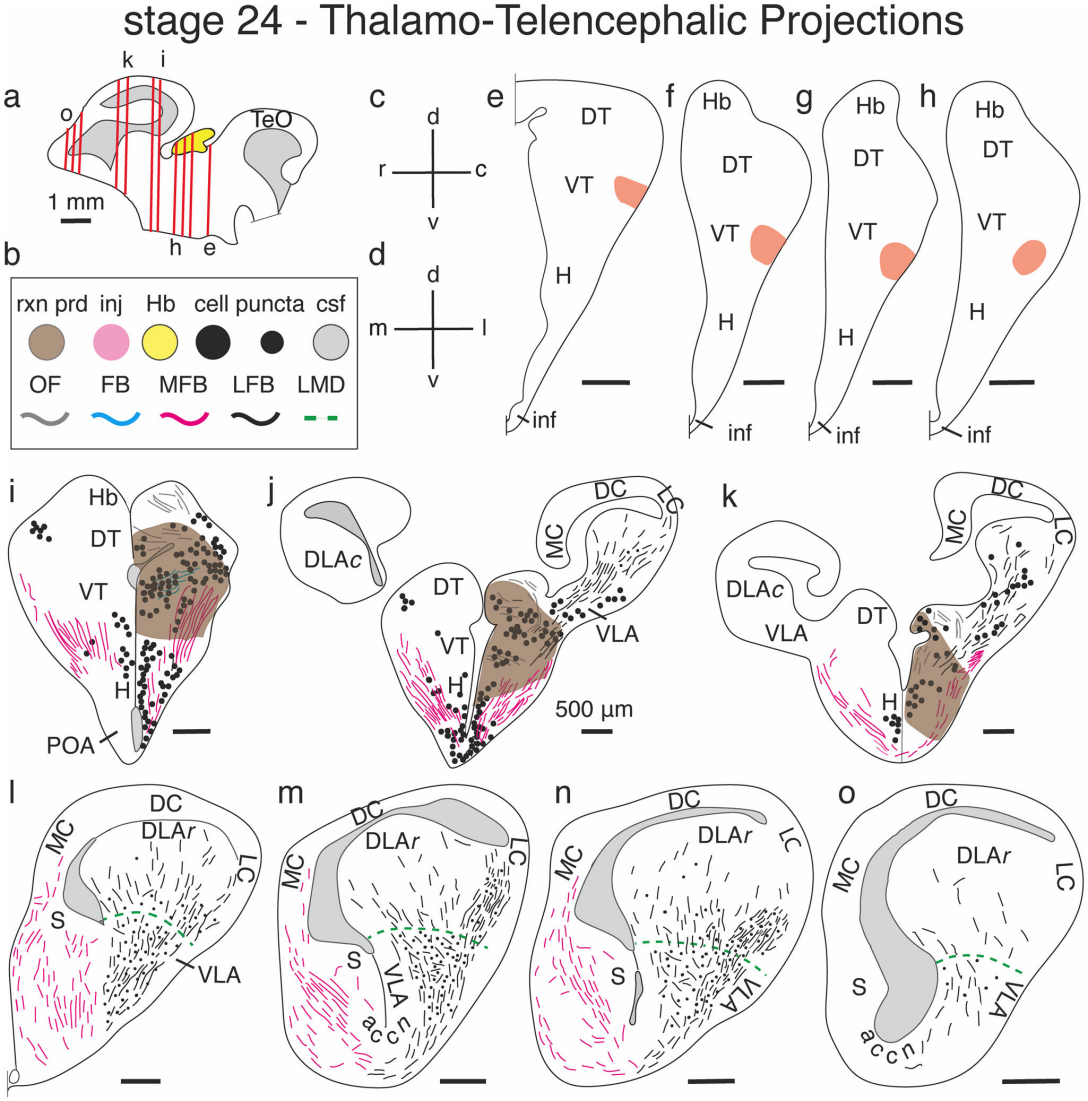
Stage 24 thalamo‐telencephalic projections. Fiber tract labeling after a tracer injection into the diencephalon is illustrated. Only the ipsilateral hemisphere was shown in the most rostral sections (l–o) because no staining was seen contralaterally. The injection site (e–h) and brown reaction product (i–k) are shown. The approximate location and plane of section for these transverse sections (e–o) are indicated on a schematic parasagittal outline of a Stage 24 embryo (a). Bar scale for each line drawing is the same and is indicated (j). The orientation for the sagittal schematic (a) is indicated (c). The orientation for the transverse sections is shown (d). Coding scheme for the transverse section line drawings is indicated (b). Abbreviations: acc*n*, accumbens nucleus; c, caudal; csf, cerebrospinal fluid; d, dorsal; DC, dorsal cortex; DLA*c*, caudal dorsolateral area; DLA*r*, rostral dorsolateral area; DT, dorsal thalamus; FB, forebrain bundle; H, hypothalamus; Hb, habenula; inf, infundibulum; inj, injection site; l, lateral; LC, lateral cortex; LFB, lateral forebrain bundle; LMD, dorsal medullary lamina; m, medial; MC, medial cortex; MFB, medial forebrain bundle; OF, other fibers; POA, preoptic area; r, rostral; rxn prd, brown reaction product; S, septum; TeO, optic tectum; v, ventral; VLA, ventrolateral area; VT, ventral thalamus (prethalamus).

**FIGURE 6:**
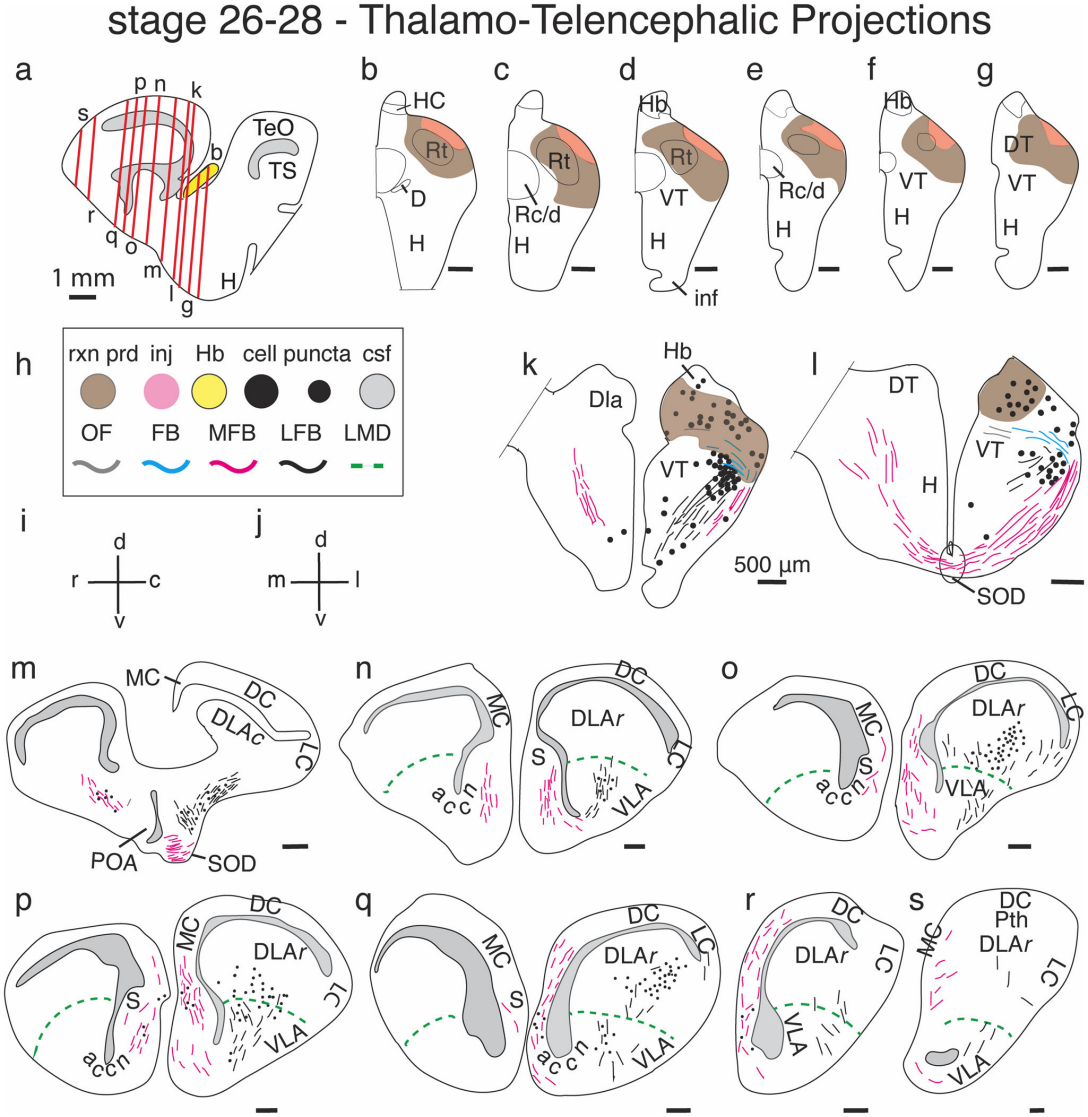
Stages 26–28 thalamo‐telencephalic projections. Fiber tract labeling after a tracer injection into the diencephalon is illustrated. Only the ipsilateral hemisphere was shown in the most rostral sections (r, s) because no staining was seen contralaterally. The injection site (b–g) and brown reaction product (b–g, k, l) are shown. The approximate location and plane of section for these transverse sections (b–g, k–s) are indicated on a schematic parasagittal outline of a Stages 26–28 embryo (a). Individual sections, c‐f, are not marked on the schematic but are equally spaced in the area represented by b–g on the schematic (a). Bar scale for each line drawing is the same and is indicated (k). The orientation for the sagittal schematic (a) is indicated (i). The orientation for the transverse sections is shown (j). Coding scheme for the transverse section line drawings is indicated (h). acc*n*, accumbens nucleus; c, caudal; csf, cerebrospinal fluid; d, dorsal; D, nucleus diagonalis; DC, dorsal cortex; Dla, nucleus dorsolateralis anterior; DLA*c*, caudal dorsolateral area; DLA*r*, rostral dorsolateral area; DT, dorsal thalamus; FB, forebrain bundle; H, hypothalamus; Hb, habenula; HC, habenular commissure; inf, infundibulum; inj, injection site; l, lateral; LC, lateral cortex; LFB, lateral forebrain bundle; m, medial; LMD, dorsal medullary lamina; MC, medial cortex; MFB, medial forebrain bundle; OF, other fibers; POA, preoptic area; Pth, pallial thickening; r, rostral; rxn prd, brown reaction product; Rc/d, nucleus reuniens pars centralis and diffusa; Rt, nucleus rotundus; S, septum; SOD, supraoptic decussation; TeO, optic tectum; TS, torus semicircularis; v, ventral; VLA, ventrolateral area; VT, ventral thalamus (prethalamus).

**FIGURE 7:**
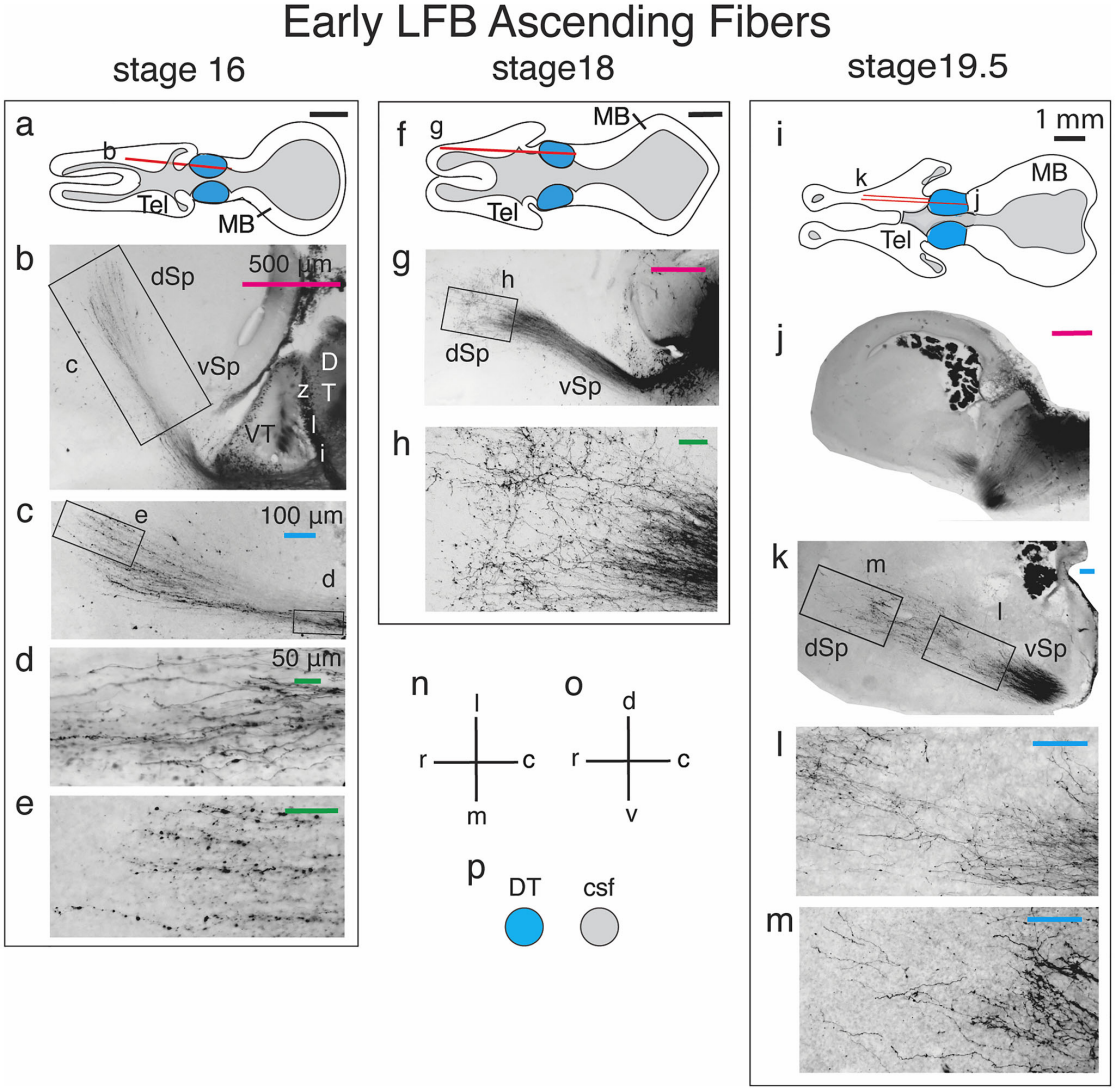
Ascending lateral forebrain bundle fibers at early developmental stages. The morphology of ascending lateral forebrain bundle fibers at Stages 16 to 19.5 is shown in sagittal sections (b‐e; g, h; j–m). The approximate location and plane of section are illustrated on horizontal schematic line drawings at stages 16 (a), 18 (f), and 19.5 (i). Enclosed boxes of lower magnification views at stages 16 (b), 18 (g), and 19.5 (k) show the origin of fibers from the dorsal thalamus that ascend to the ipsilateral hemisphere. The morphology of fibers are shown at higher magnification at stage 16 (c–e), 18 (h), and 19.5 (l, m). Orientation for the horizontal schematic is indicated (n). The color coding scheme is shown (p). Similar color coding for bar scales reflects identical lengths. c, caudal; csf, cerebrospinal fluid; d, dorsal; dSp, dorsal subpallium; DT, dorsal thalamus; l, lateral; m, medial; MB, midbrain; r, rostral; Tel, telencephalon; v, ventral; vSp, ventral subpallium; VT, ventral thalamus (prethalamus); zli, zona limitans intraparencephalica.

**FIGURE 8:**
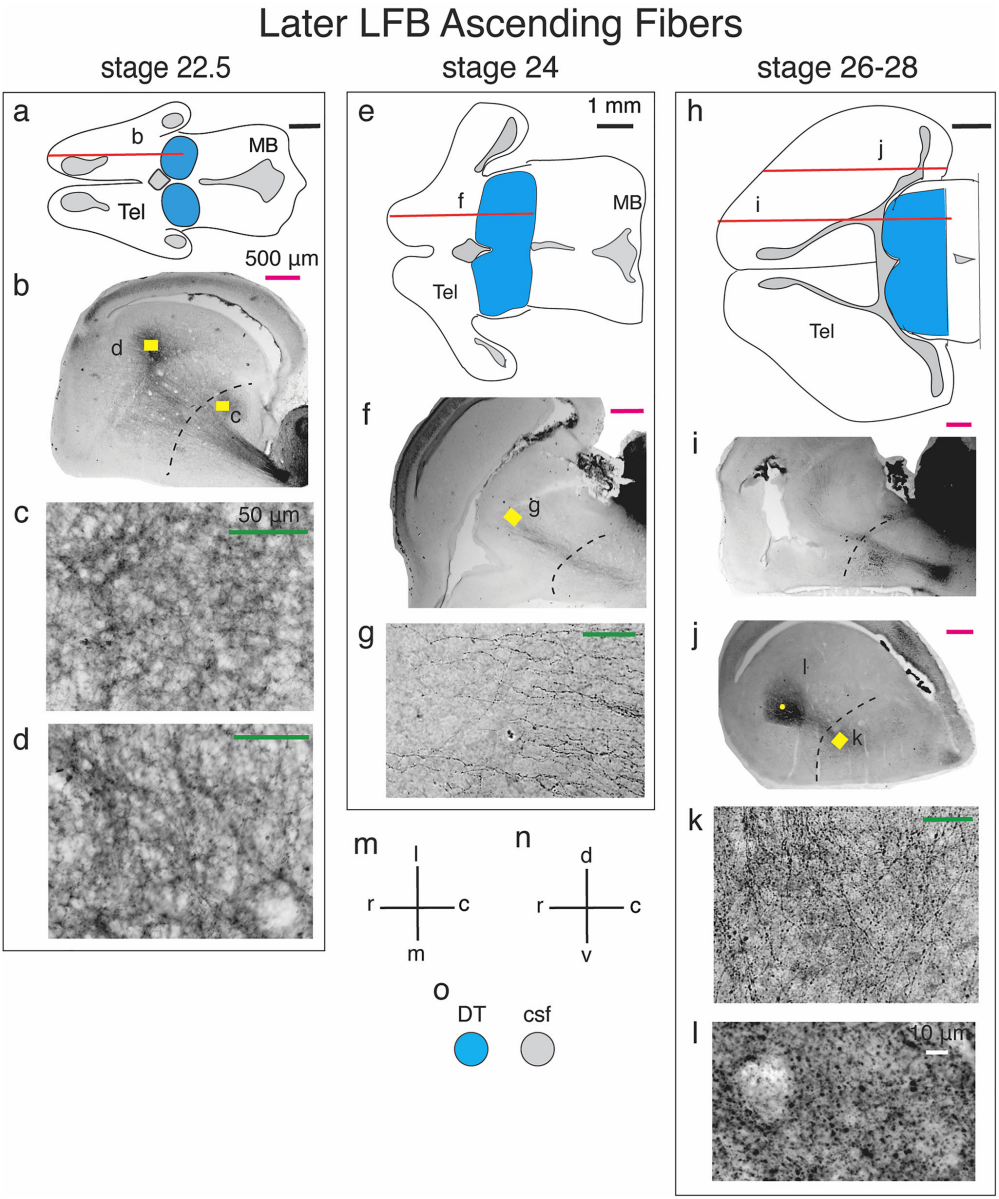
Ascending lateral forebrain bundle fibers at later developmental stages. The morphology of ascending lateral forebrain bundle fibers at Stages 22.5 to 26–28 is shown in sagittal sections (b–d; f, g; i–l). The approximate location and plane of section are illustrated on horizontal schematic line drawings at Stages 22.5 (a), 24 (e), and 26–28 (h). Enclosed boxes of lower magnification views at stages 22.5 (b), 24 (f), and 26–28 (j) show the trajectory of fibers from the dorsal thalamus ascending to the ipsilateral hemisphere. The morphology of fibers and puncta are shown at higher magnification at stage 22.5 (c, d), 24 (g), and 24–26 (k, l). Orientation for the horizontal schematic is indicated (m). The orientation of the sagittal photos is shown (n). The color coding scheme is indicated (o). Broken lines (b, f, i) represent the approximate location of the dorsal medullary lamina. Similar color coding for bar scales reflects identical lengths. c, caudal; csf, cerebrospinal fluid; d, dorsal; DT, dorsal thalamus; l, lateral; m, medial; MB, midbrain; r, rostral; Tel, telencephalon; v, ventral.

The sequential development and associated terminology of dorsal thalamic and telencephalic nuclei and forebrain tracts have been described (Pritz 2025a, 2025b, 2025c). Within these papers were details of the morphology of these areas between Stages 16 and 26–28. Although these findings have been reported previously, a brief summary of these prior observations is provided to serve as a background for understanding the results of the present experiments. In addition, because the morphology of individual stages between Stages 26 and 28 was so similar, Stages 26–28 were treated as a single unit.

Early in development, between Stages 16 and 20, borders of nuclei in the diencephalon were based on the following features in transverse sections (Pritz 2025a). The habenula (Pritz 2025a) and the infundibulum (Subhedar et al. 1989) were identified by their respective external shape (Figures 1d‐g, 2h‐i, and 3e–i). Early in development, at Stage 16, the dorsal thalamus was marked by fibers exiting ventrally (Figure 7b,g), whereas the ventral thalamus (prethalamus; Figures 2h and 3j) contained retrogradely labeled cells in the midst of the forebrain bundle fibers. The hypothalamus was considered as the area between the ventral (prethalamus) thalamus and the infundibulum (Figures 1d–h, 2h, and 3e‐i. The preoptic area was identified by its location, surrounding the preoptic recess (Figures 2i–l and 3g–i,l). In the telencephalon, the region dorsal and external to the lateral ventricle was the pallium (Figures 1n–p, 2i–n, and 3l–q). At this time in development, the area ventral to the pallium was the subpallial area that had a dorsal and ventral component (Figures 1n–q, 2i–n, and 3l–q). The dorsal subpallium became the dorsolateral area (dorsal ventricular ridge of other sauropsids), whereas the ventral subpallium developed into the ventrolateral area (part of the basal nuclei). These terms, dorsal and ventral subpallium, were anatomical descriptors that were used to identify these structures at Stages 16–20. As such, they were not meant to indicate their future status. Specifically, the dorsolateral area in crocodiles and the dorsal ventricular ridge in other reptiles are pallial structures (Medina et al. 2021). The septum was identified as a bulge between the pial surface medially and the lateral ventricle laterally (Figures 2l–n and 3n–q). The accumbens area was the region between the septum and the ventral subpallium (Figures 2l–n and 3n–q; Pritz 2025b, 2025c).

Later in development, between Stages 22 and 26–28, features described previously were used to identify nuclei (Pritz 2025c). In addition, some modifications were used in the present analysis for the following reasons. In the telencephalon, at Stage 22, anlage of the divisions of the medial cortex were present (Figures 4k–r; Pritz 2025c). However, at this stage and later, these were treated as a single unit because cytoarchitectonic divisions could not be reliably distinguished in the present material. In addition, the ventrolateral area (Figures 4k–p, 5j–o, and 6n–s) was not subdivided into dorsal and ventral parts for similar reasons. The divisions of the dorsolateral area were based on the presence or the absence of the dorsal medullary lamina. When the dorsal medullary lamina was present, the rostral dorsolateral area (Figures 4m–p; 5l–o, and 6n–s) was noted. However, when the dorsal medullary lamina was absent, this defined the caudal dorsolateral area (Figures 4k,l, 5j,k, and 6m; Pritz 2025c). The dorsolateral area of *Alligator* corresponded to the dorsal ventricular ridge of other reptiles. Originally (Pritz 2025c), the ventrolateral area was considered the sole structure termed the basal nuclei. However, the present observations indicated that the structures labeled as the accumbens nucleus and the septum should be included as parts of the basal nuclei at Stages 22 to 26–28. Similarly, at Stages 16–20, the structures marked as septum, accumbens area, and ventral subpallium comprised the basal nuclei.

In the diencephalon, nucleus rotundus was identified based on its characteristic shape at stage 22 (Figure 4e,f). However, its borders were not distinct until later in development at Stage 23 when surrounding nuclei could be identified. For this reason, at Stage 22, nucleus rotundus was considered to be its anlage. A distinction between the pars centralis and pars diffusa of nucleus reuniens could not be made (Figure 6b–f). For this reason, these two nuclei were referred to as the nucleus reuniens complex.

A brown reaction product surrounded the neurobiotin injection site. The size and extent of this brown reaction product varied and, in some instances, it extended well beyond the injection site itself. This is discussed further (Discussion 4.1).

### 3.2 Circuit Formation and Development

Neurobiotin injections into the diencephalon identified both anterograde fibers and presumed terminals as well as retrogradely labeled cells. This allowed for identification of four circuits that interconnected the dorsal thalamus with the telencephalon. These circuits were the ipsilateral lateral forebrain bundle and the ipsilateral and contralateral medial forebrain bundle. The fourth circuit was identified by retrograde labeling of cells in the basal nuclei that projected axons to the diencephalon.

In order to follow the development of anterogradely labeled axons, fibers were color‐coded in transverse sections (Figures 1, 2, 3, 4, 5, 6). Four groups were marked. These were: forebrain bundle, lateral forebrain bundle, medial forebrain bundle, and “other fibers.” The forebrain bundle referred to those fibers that exited the diencephalon as a compact bundle before dividing into the lateral and medial forebrain bundles. The category termed other fibers included labeled axons in the forebrain not associated with either the forebrain, lateral forebrain, or medial forebrain bundles.

Two other types of data were included to support these observations. One was the use of selected sagittal photos (Figures 7 and 8), which better visualized the entirety of the lateral forebrain bundle anterograde path than transverse sections. The other detailed the appearance of presumed synapse formation (Figure 9) by comparing puncta observed after anterograde tracer injections with the expression of SV2.

**FIGURE 9:**
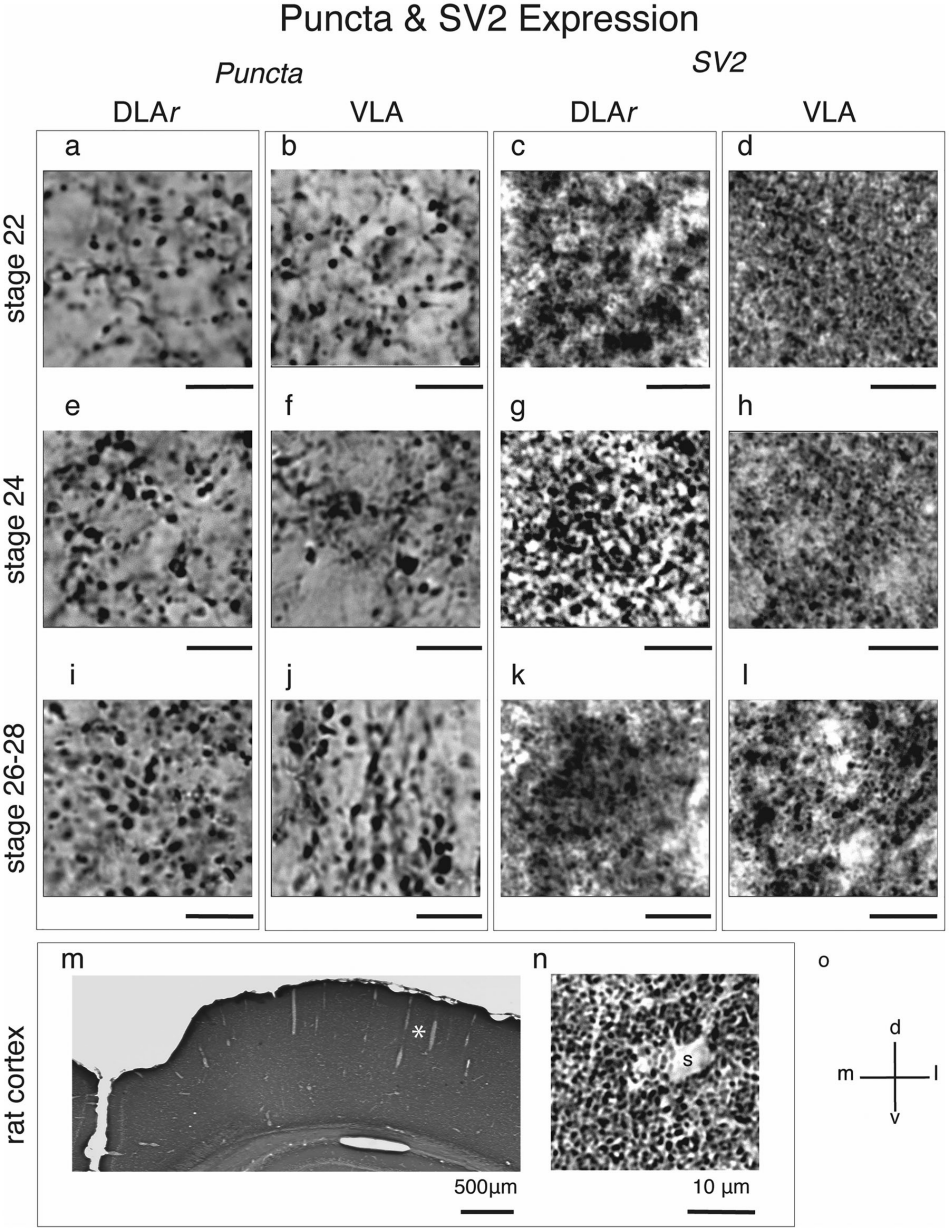
Puncta and SV2 expression. The appearance of puncta and SV2 expression in the rostral dorsolateral and ventrolateral areas is shown (a–l). A positive control from a section of rat forebrain (m, n) demonstrates the morphology of SV2 elements surrounding a neuron (S). This higher magnification image (n) is from the area marked by an asterisk (*) in m. Bar scale for all high magnification photos is the same and is marked (n). Orientation of the transverse sections is shown (o). d, dorsal; DLA*r*, rostral dorsolateral area; l, lateral; m, medial; S, soma; SV2, synaptic vesicle 2; v, ventral; VLA, ventrolateral area.

### 3.3 Development of Lateral and Medial Forebrain Circuits

Following tracer injections into the diencephalon, fibers along the lateral edge of the diencephalon divided into two bundles. On their way to the telencephalon, the lateral forebrain bundle remained at the mid‐dorsoventral level, while the medial forebrain bundle reached the ventral part of the diencephalon. The further development of each of these tracts is described below.

#### 3.3.1 Lateral Forebrain Bundle Projections to the Telencephalon

The course of ipsilateral fibers and terminals observed after tracer injections into the forebrain bundles or diencephalon between Stages 16 and 26–28 were illustrated (Figures 1, 2, 3, 4, 5, 6, 7, 8). These data were shown from two perspectives: transverse and sagittal sections. The former (Figures 1, 2, 3, 4, 5, 6) was best suited for tracing the course of the lateral forebrain bundle through the diencephalon to reach its terminus in the telencephalon.

The latter (Figures 7 and 8) provided an overall view of the course of this tract.

##### 3.3.1.1 Stage 16

Before Stage 19.5, both the dorsal thalamus and the telencephalon (with the exception of the septum) remain undifferentiated. Nevertheless, fibers leaving the diencephalon reached the telencephalon at this earliest of stages. Labeling of lateral forebrain bundle fibers resulting from a diencephalic tracer injection was shown in transverse sections (Figure 1). The injection site was located in a lateral part of the dorsal thalamus and labeled the forebrain bundles (Figure 1d–f). Brown reaction product (Figure 1d–g) abutted the injection site (Figure 1d–f) and extended further rostrally (Figure 1g). Fibers coursed medially to leave the diencephalon (Figure 1d) to reach the ventral subpallium (Figure 1n–p). This overall trajectory was better seen in sagittal images than was observed in transverse sections. From this sagittal perspective, fibers left the dorsal thalamus, coursed ventral to the ventral thalamus (prethalamus) to reach the telencephalon (Figure 7b). This basic pattern of lateral forebrain bundle organization served as a framework for all subsequent stages. At this time, very fine axons were observed that reached the dorsal subpallium (Figure 7b, c, e) where separate fascicles were seen (Figure 7e).

##### 3.3.1.2 Stage 18

At Stage 18, the lateral forebrain circuit was better defined in transverse sections (Figure 2). The injection site labeled the dorsal thalamus and the more laterally located exiting fiber bundle (Figure 2e–g). Axons left the forebrain bundle, entered the lateral forebrain bundle, coursed medially (Figure 2h), ascended through the accumbens area (Figure 2i,j), traversed the ventral subpallial area (Figure 2j–n), and a few scattered fibers reached the overlying dorsal subpallial area (Figure 2k–n). In sagittal sections, labeled axons from an injection into the diencephalon coursed ventrally and traveled as a distinct bundle to reach the telencephalon (Figure 7g,h).

##### 3.3.1.3 Stages 19.5 and 20

Lateral forebrain bundle fibers observed after a tracer injection were shown in transverse sections (Figure 3). The injection site labeled a lateral portion of the dorsal thalamus and the adjacent forebrain bundle (Figure 3e–i). The trajectory and appearance of labeled fibers at this stage were similar to stage 18 except for a greater number of axons that were observed in the ventral and dorsal subpallium (Figure 3m–q). Similarly, the morphology of fibers and their manner of termination were comparable to that seen at stage 18 (Figure 7k–m).

##### 3.3.1.4 Stages 22 and 22.5

At Stages 22 and 22.5, several changes in the forebrain occurred. First, the dorsal thalamus and the telencephalon underwent further differentiation. Second, the dorsal medullary lamina, which separated the rostral dorsolateral area (dorsal ventricular ridge) from the ventrolateral area, was identifiable. Third, puncta were seen. The distribution of labeled fibers, cells, and puncta after an injection into the dorsal thalamus (Figure 4d–g) that mainly involved the intermediate tier, which included part of the anlage of nucleus rotundus (Figure 4e,f), is illustrated. The brown reaction product observed subsequent to this injection extended a considerable distance rostrally (Figure 4i–k).

While the overall course and trajectory of the lateral forebrain bundle were similar to prior stages, axons and their terminations, based on the appearance of puncta, became more distinct (Figures 4l–p and 8b). For one, fibers entering the dorsal peduncle of the lateral forebrain bundle were clearly seen (Figure 4i,j). Terminal fields in the dorsolateral area, reminiscent of the projection fields of sensory thalamic nuclei in juvenile crocodilians (Pritz 1974, 1975; Pritz and Stritzel 1994), were observed (Figures 4n,o and 8b). Moreover, puncta were found throughout additional parts of the dorsolateral and ventrolateral areas (Figure 4l‐p). In addition, for the first time during the course of development, retrogradely labeled cells were observed within the fibers of the lateral forebrain bundle (Figure 4k–m).

##### 3.3.1.5 Stages 24 and 26–28

The course and trajectory of the lateral forebrain bundle at these two later stages in development (Figures 5 and 6) were similar to what was found at stage 22 with the following additions. One was the greater number of puncta in the ventrolateral and dorsolateral areas (Figures 5l–o and 6o–q). A second was an increased number of fibers and their thickness observed in the telencephalon (Figures 5j–o, 6n–s, and 8g,j). Third, a larger number of retrogradely labeled cells within the fascicles of the lateral forebrain bundle was seen (Figures 5j,k and 6m,n). Last, at Stages 26–28, labeled fibers extended further anteriorly reaching the rostral pole of the telencephalon (Figure 6r,s).

#### 3.3.2 Medial Forebrain Bundle Projections to the Telencephalon

The course and trajectory of ipsilateral and contralateral fibers in the medial forebrain bundles were illustrated in a series of transverse sections between Stages 16 and 26–28 (Figures 1, 2, 3, 4, 5, 6). Included were photos of these fibers at the earliest of these stages, Stage 16 (Figure 1).

##### 3.3.2.1 Stage 16

Labeling of medial forebrain bundle fibers resulting from a diencephalic tracer injection was shown in transverse sections (Figure 1). The injection site was located in a lateral part of the dorsal thalamus and labeled the forebrain bundles (Figure 1d–f). Brown reaction product (Figure 1d–g) abutted the injection site (Figure 1d–f) and extended further rostrally (Figure 1g). Labeled fibers collected on the lateral border of the diencephalon and coursed ventrally (Figure 1f–h,k–m). Rostrally, fibers continued ventrally along the lateral wall (Figure 1g,h,l,m). These ascending fibers reached the supraoptic decussation and barely extended to the opposite side (Figure 1h,m).

##### 3.3.2.2 Stage 18

The distribution of fibers resulting from an injection that labeled the dorsal thalamus and the more laterally located exiting fiber bundle was shown (Figure 2e–g). Ascending fibers collected along the lateral edge of the diencephalon and coursed ventrally (Figure 2h,i). At this level, this fiber bundle split into two: one traveled ipsilaterally while the other went contralaterally. The ipsilateral component coursed rostrally and turned medially to ascend along the medial aspect of the hemisphere (Figure 2m, n). The contralateral component continued ventrally, entered and passed through the supraoptic decussation, and crossed to the opposite side (Figure 2j–l) where rare fibers were seen in the medial hemisphere. (Figure 2m,n).

##### 3.3.2.3 Stage 20

Medial forebrain bundle fibers observed after a tracer injection in a Stage 20 embryo were shown in transverse sections (Figure 3). The injection labeled a lateral portion of the dorsal thalamus and the adjacent forebrain bundle (Figure 3e–i). Similar to Stage 18, fibers in the medial forebrain bundle split into two tracts. The ipsilateral component continued rostrally and turned dorsally to enter the medial hemisphere passing through the septum (Figure 3n–q). The contralateral bundle passed through the supraoptic decussation (Figure 3k) to reach the opposite hemisphere (Figure 3l–o). No staining was observed further rostrally in the contralateral hemisphere in this case. For this reason, only ipsilateral hemisphere labeling was shown for the two most rostral sections (Figure 3p,q).

##### 3.3.2.4 Stages 22 to 26–28

The course and termination of the ipsilateral and contralateral medial forebrain bundle axons followed the same pattern described for the earlier developmental times. The injection locus and the trajectory of the medial forebrain tract were shown (Figures 4, 5, 6). Although the overall course and distribution of fibers followed a similar plan, several important changes occurred. First, the number and thickness of labeled axons increased as development proceeded. Second, patches of puncta that were prominent in the dorsolateral area after injections that labeled ascending lateral forebrain bundle fibers were not seen. Third, fibers that ascended along the medial wall of the hemisphere never reached that part of the medial cortex that abutted the dorsal cortex. Fourth, fiber number and extent along the medial hemisphere were greater ipsilaterally than contralaterally.

### 3.4 Puncta and Synaptic Vesicle 2 (SV2) Expression

At stage 20, SV2 immunostaining was seen in the optic tectum and hypothalamus but not in the basal nuclei or rostral dorsolateral area. Patches of labeled puncta and dispersed puncta in the forebrain were only observed at Stage 22 and thereafter. Their appearance at Stages 22 to 26–28 was shown in transverse sections in both the rostral dorsolateral and ventrolateral areas (Figure 9a,b,e,f,i,j). A similar appearance of puncta was likewise observed in sagittally sectioned material (Figures 8c,d,k,l). To support the suggestion that puncta represented synaptic sites, SV2 immunostaining was conducted. SV2 labeling was not observed in the forebrain before Stage 22. From Stage 22 onward, SV2 labeling showed similar morphology to puncta and was located in the same forebrain regions where patches of puncta were observed after tracer application to the diencephalon (Figure 9c,d,g,h,k,l). The morphology of labeled elements observed in SV2‐stained material in *Alligator* forebrain was similar to that seen in similarly treated sections of rat cortex processed in the same fashion and with same concentration of primary antibody (Figure 9n).

### 3.5 Basal Nuclei Projections to the Diencephalon

Retrogradely labeled cells in the basal nuclei after tracer injections into the diencephalon and forebrain bundles were examined in transverse sections. Observations began with Stage 16 and continued through Stages 26–28 (Figures 10, 11, 12, 13, 14, 15).

**FIGURE 10:**
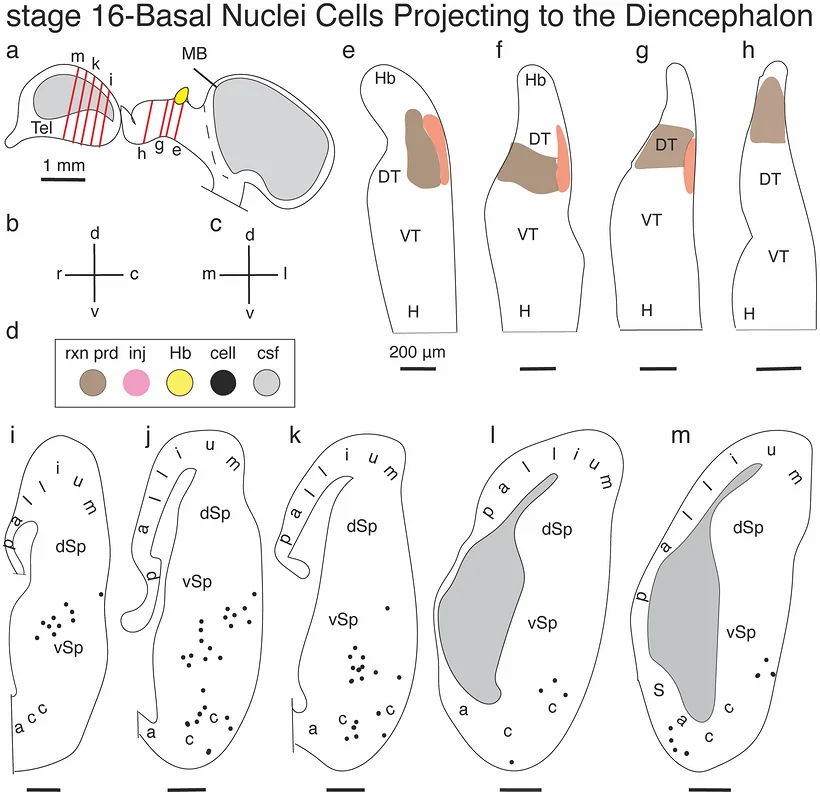
Stage 16 basal nuclei cells projecting to the diencephalon. Transverse sections show the distribution of retrogradely labeled cells in the basal nuclei after a diencephalic tracer injection. The injection locus and the brown reaction product are indicated (e–h). Retrogradely labeled cells in the basal nuclei are shown (i–m). The approximate location and plane of section of these transverse sections are marked on a sagittal schematic of a Stage 16 embryo (a). The orientation of the sagittal schematic (b) and transverse sections (c) is shown. The coding scheme is indicated (d). acc, accumbens area; c, caudal; csf, cerebrospinal fluid; d, dorsal; dSp, dorsal subpallium; DT, dorsal thalamus; H, hypothalamus; Hb, habenula; inj, injection site; l, lateral; m, medial; MB, midbrain; r, rostral; rxn prd, brown reaction product; S, septum; v, ventral; vSp, ventral subpallium; VT, ventral thalamus (prethalamus).

**FIGURE 11:**
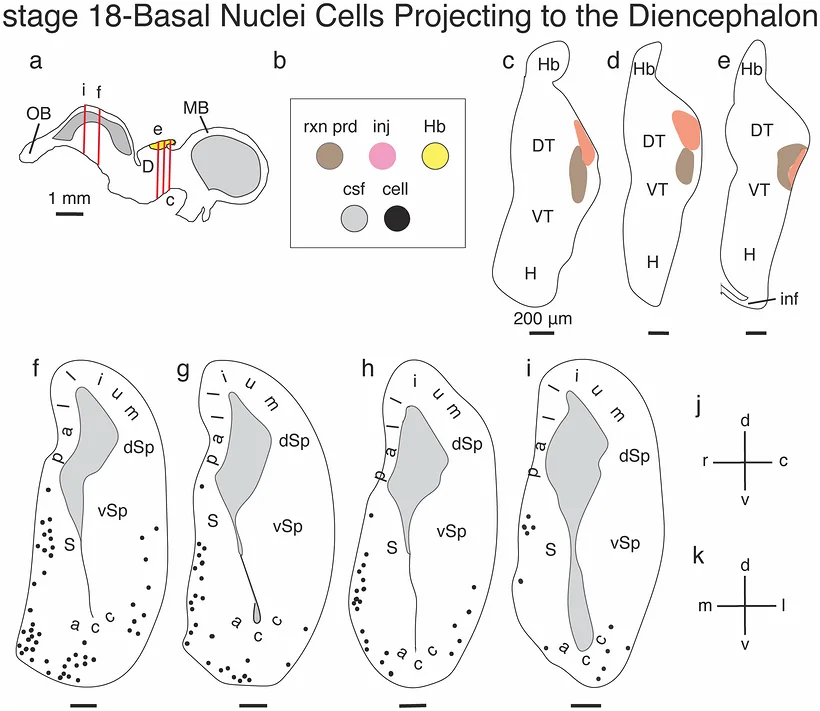
Stage 18 basal nuclei cells projecting to the diencephalon. Transverse sections show the distribution of retrogradely labeled cells in the basal nuclei after a diencephalic tracer injection. The injection locus and the brown reaction product are indicated (c–e). Retrogradely labeled cells in the basal nuclei are shown (f–i). The approximate location and plane of section of these transverse sections are marked on a sagittal schematic of a Stage 18 embryo (a). The orientation of the sagittal schematic (j) and transverse sections (k) is shown. The coding scheme is indicated (b). acc, accumbens area; c, caudal; csf, cerebrospinal fluid; d, dorsal; D, diencephalon; dSp, dorsal subpallium; DT, dorsal thalamus; H, hypothalamus; Hb, habenula; inf, infundibulum; inj, injection site; l, lateral; m, medial; MB, midbrain; OB, olfactory bulb; r, rostral; rxn prd, brown reaction product; S, septum; v, ventral; vSp, ventral subpallium; VT, ventral thalamus (prethalamus).

**FIGURE 12:**
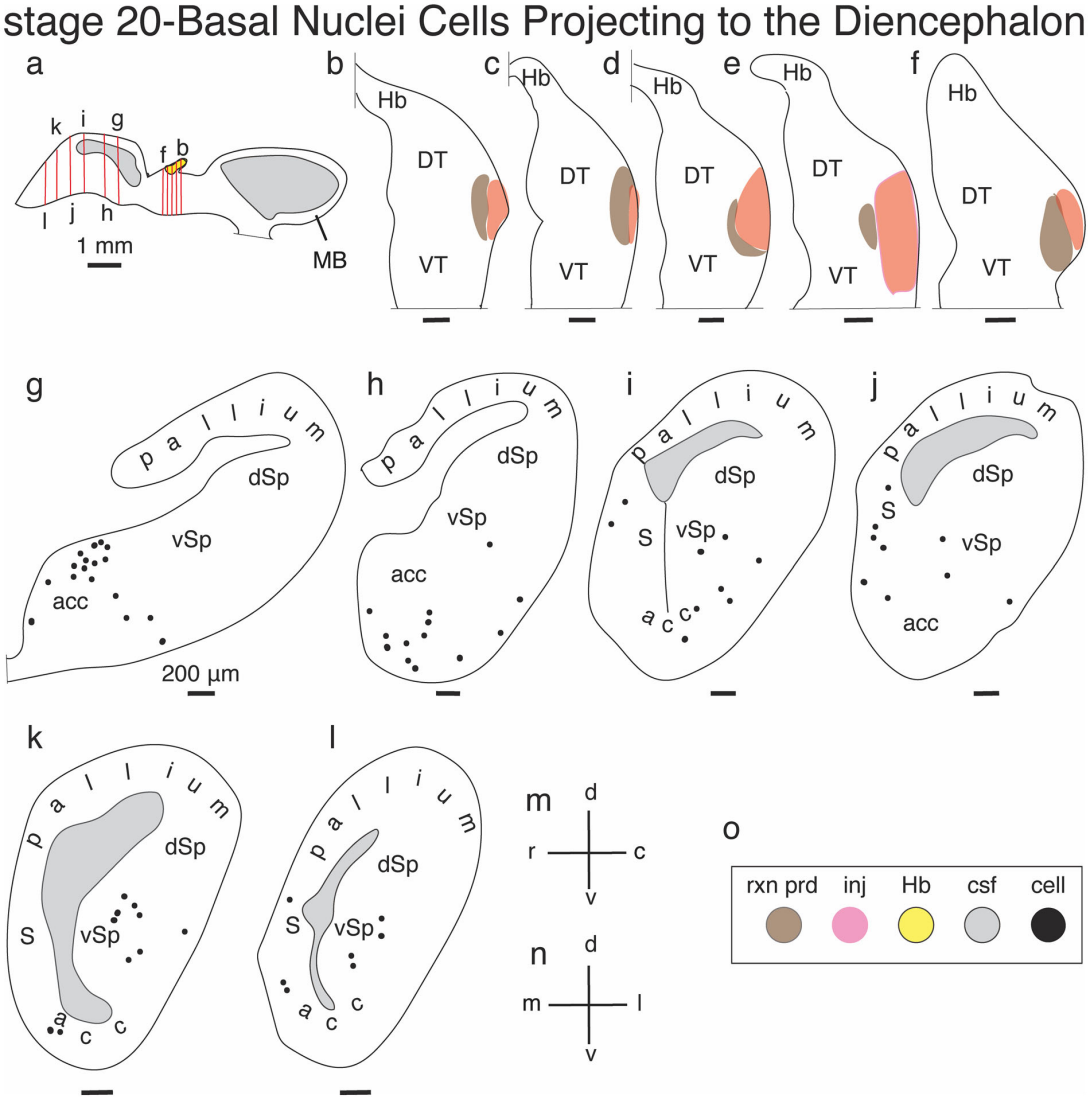
Stage 20 basal nuclei cells projecting to the diencephalon. Transverse sections show the distribution of retrogradely labeled cells in the basal nuclei after a diencephalic tracer injection. The injection locus and the brown reaction product are indicated (b–f). Retrogradely labeled cells in the basal nuclei are shown (g–l). The approximate location and plane of section of these transverse sections are marked on a sagittal schematic of a stage 20 embryo (a). The orientation of the sagittal schematic (m) and transverse sections (n) is shown. The coding scheme is indicated (o). acc, accumbens area; c, caudal; csf, cerebrospinal fluid; d, dorsal; dSp, dorsal subpallium; DT, dorsal thalamus; H, hypothalamus; Hb, habenula; inj, injection site; l, lateral; m, medial; MB, midbrain; r, rostral; rxn prd, brown reaction product; S, septum; v, ventral; vSp, ventral subpallium; VT, ventral thalamus (prethalamus).

**FIGURE 13:**
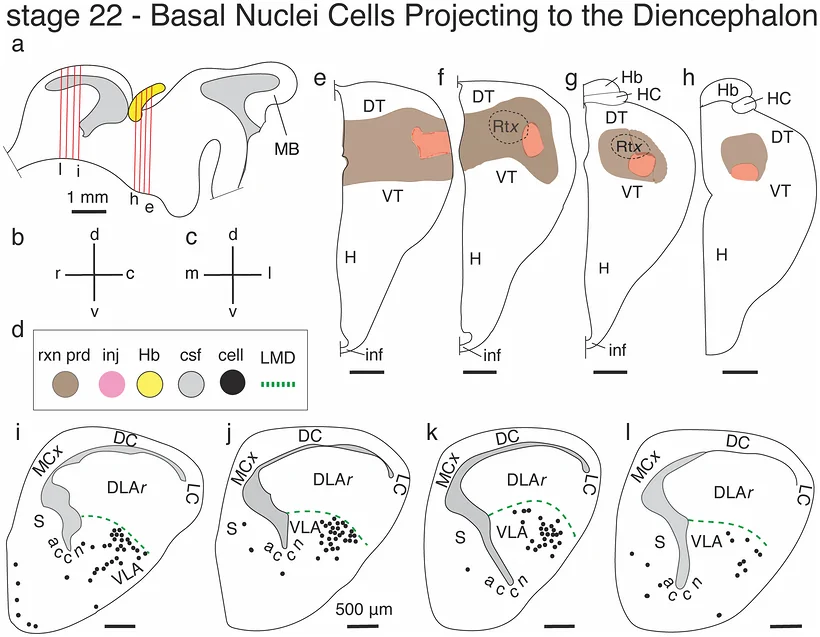
Stage 22 basal nuclei cells projecting to the diencephalon. Transverse sections show the distribution of retrogradely labeled cells in the basal nuclei after a diencephalic tracer injection. The injection locus and the brown reaction product are indicated (e‐h). Retrogradely labeled cells in the basal nuclei are shown (i‐l). The approximate location and plane of section of these transverse sections are marked on a sagittal schematic of a stage 22 embryo (a). The orientation of the sagittal schematic (b) and transverse sections (c) is shown. The coding scheme is indicated (d). acc*n*, accumbens nucleus; c, caudal; csf, cerebrospinal fluid; d, dorsal; DC, dorsal cortex; DLA*r*, rostral dorsolateral area; DT, dorsal thalamus; H, hypothalamus; Hb, habenula; HC, habenular commissure; inf, infundibulum; inj, injection site; l, lateral; LC, lateral cortex; LMD, dorsal medullary lamina; m, medial; MB, midbrain; MC*x*, medial cortex anlage; r, rostral; rxn prd, brown reaction product; Rt*x*, nucleus rotundus anlage; S, septum; v, ventral; VLA, ventrolateral area; VT, ventral thalamus (prethalamus).

**FIGURE 14:**
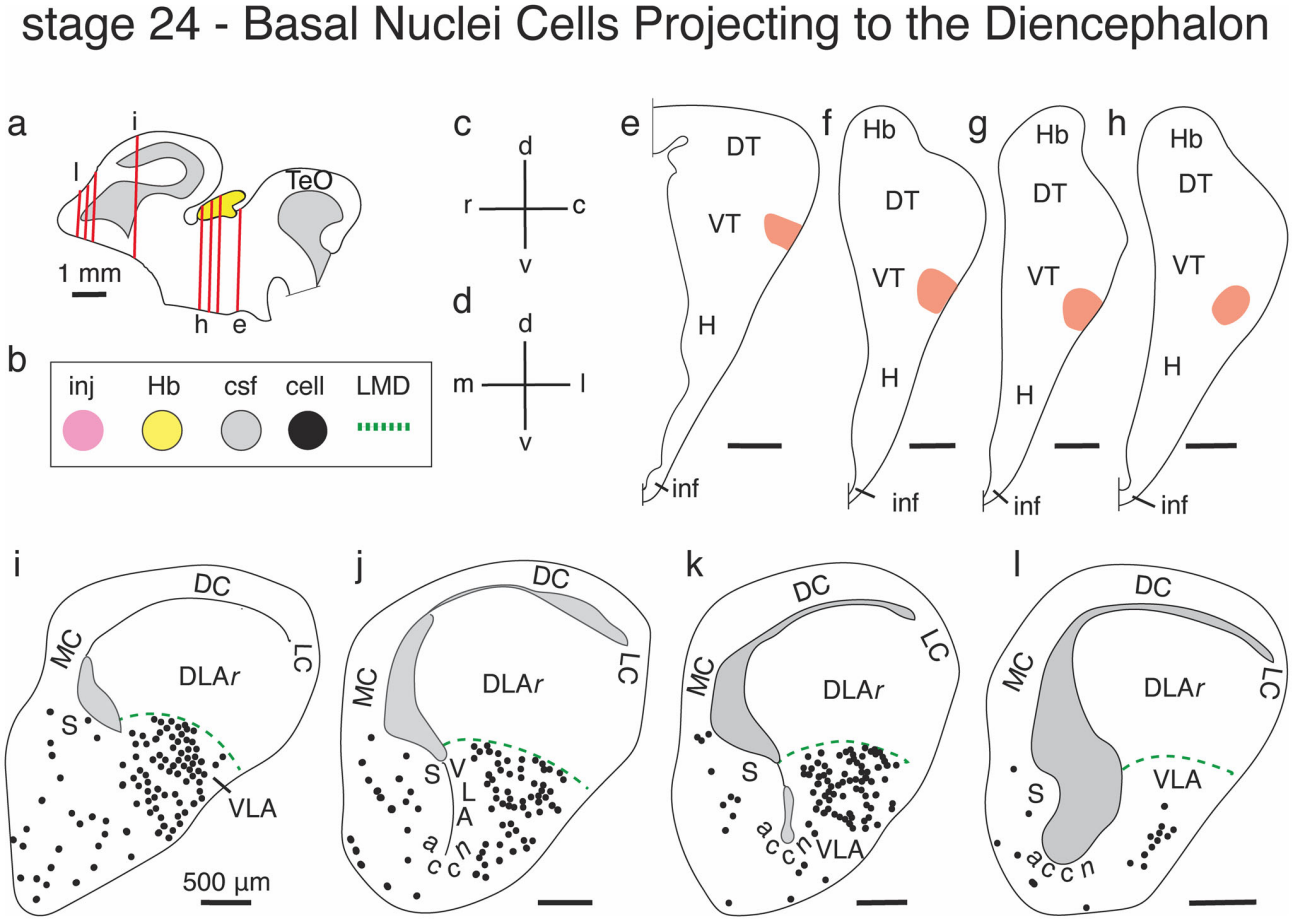
Stage 24 basal nuclei cells projecting to the diencephalon. Transverse sections show the distribution of retrogradely labeled cells in the basal nuclei after a diencephalic tracer injection. The injection locus is indicated (e–h). Retrogradely labeled cells in the basal nuclei are shown (i–l). The approximate location and plane of section of these transverse sections are marked on a sagittal schematic of a Stage 24 embryo (a). The orientation of the sagittal schematic (c) and transverse sections (d) is shown. The coding scheme is indicated (b). acc*n*, accumbens nucleus; c, caudal; csf, cerebrospinal fluid; d, dorsal; DC, dorsal cortex; DLA*r*, rostral dorsolateral area; DT, dorsal thalamus; H, hypothalamus; Hb, habenula; inf, infundibulum; inj, injection site; l, lateral; LC, lateral cortex; LMD, dorsal medullary lamina; m, medial; MC, medial cortex; r, rostral; S, septum; TeO, optic tectum; v, ventral; VLA, ventrolateral area; VT, ventral thalamus (prethalamus).

**FIGURE 15:**
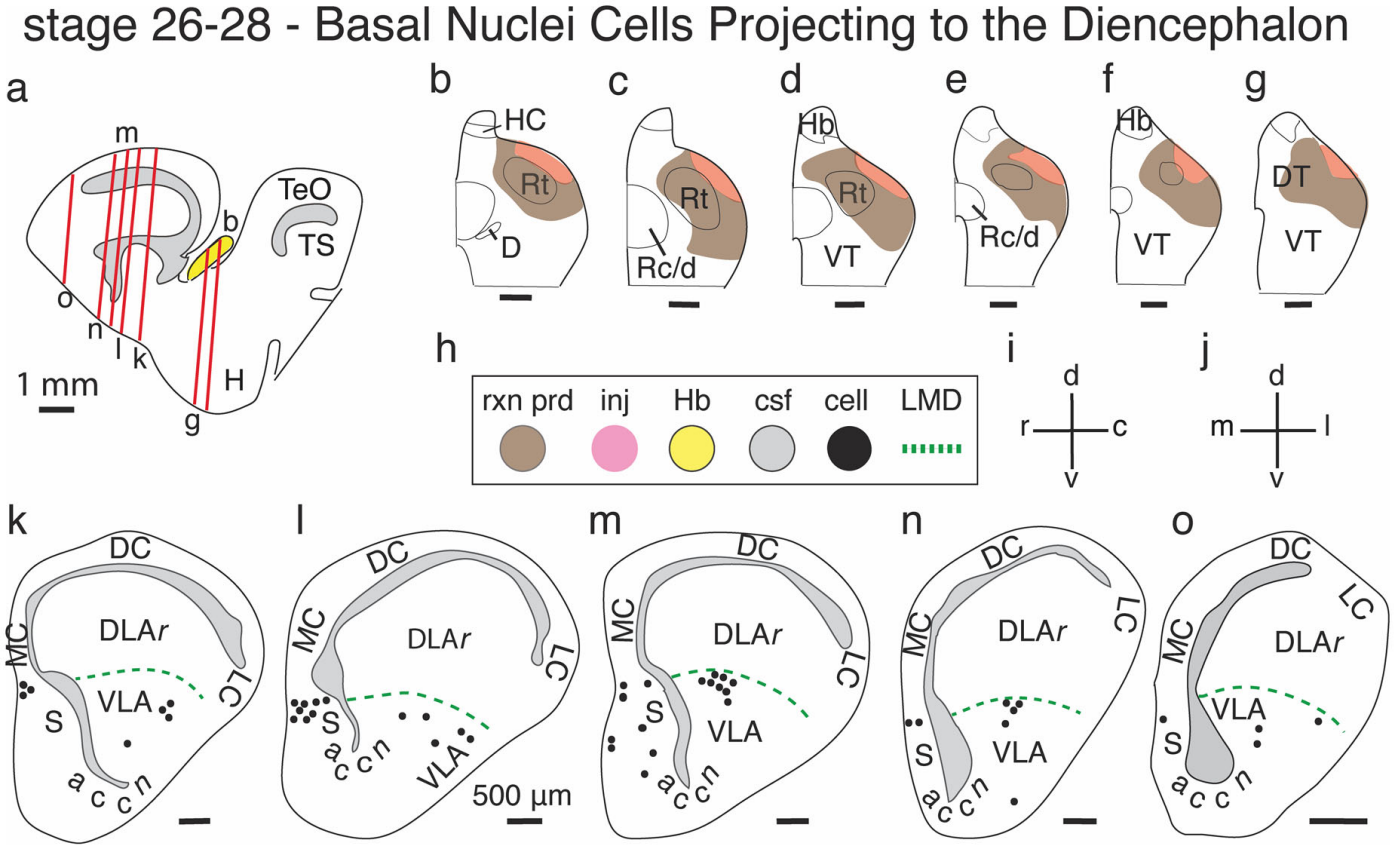
Stages 26–28 basal nuclei cells projecting to the diencephalon. Transverse sections show the distribution of retrogradely labeled cells in the basal nuclei after a diencephalic tracer injection. The injection locus is indicated (b–g). Retrogradely labeled cells in the basal nuclei are shown (k–o). The approximate location and plane of section of these transverse sections are marked on a sagittal schematic of a Stages 26–28 embryo (a). The orientation of the sagittal schematic (i) and transverse sections (j) is shown. The coding scheme is indicated (h). acc*n*, accumbens nucleus; c, caudal; csf, cerebrospinal fluid; d, dorsal; D, nucleus diagonalis; DC, dorsal cortex; DLA*r*, rostral dorsolateral area; DT, dorsal thalamus; H, hypothalamus; Hb, habenula; HC, habenular commissure; inf, infundibulum; inj, injection site; l, lateral; LC, lateral cortex; LMD, dorsal medullary lamina; m, medial; MC, medial cortex; r, rostral; Rc/d, nucleus reuniens pars centralis and pars diffusa; Rt, nucleus rotundus; rxn prd, brown reaction product; S, septum; TeO, optic tectum; TS, torus semicircularis; v, ventral; VLA, ventrolateral area; VT, ventral thalamus (prethalamus).

#### 3.5.1 Stage 16

The distribution of retrogradely labeled cells in the ipsilateral basal nuclei (Figure 10i–m) after a tracer injection into the forebrain bundles (Figure 10e–h) was shown. Labeled cells were found mainly in the ventral subpallium (Figure 10i–m) and, to a lesser extent, in the accumbens area (Figure 10j–m) in the caudal half of the telencephalon.

#### 3.5.2 Stage 18

The distribution of retrogradely labeled cells in the ipsilateral basal nuclei (Figure 11f–i) after a tracer into the forebrain bundles (Figure 11c–e) was shown. Labeled cells were found mainly in the septum and accumbens area (Figure 11f–i) and, to a lesser extent, in the ventral subpallium (Figure 11f–h).

#### 3.5.3 Stage 20

Retrogradely labeled cells in the ipsilateral basal nuclei were shown (Figure 12g–l) after a tracer injection into the diencephalon. This injection labeled the forebrain bundles and adjacent lateral dorsal thalamus (Figure 12b–f). Labeled cells were located in the accumbens area, the ventral subpallial area, and the septum (Figure 12g–l).

#### 3.5.4 Stage 22

Retrogradely labeled cells in the ipsilateral basal nuclei (Figure 13i–l) after a tracer injection into the diencephalon involving part of the anlage of nucleus rotundus (Figure 13e–h) were shown. Labeled cells were located mainly in the ventrolateral area and to a lesser extent in the septum and accumbens nucleus (Figure 13i–l) in the central part of the telencephalon (Figure 13a).

#### 3.5.5 Stage 24

Retrograde labeling of cells in the ipsilateral basal nuclei (Figure 14i–l) after an injection into the forebrain bundles (Figure 14e–h) was shown. Labeled cells were located throughout the basal nuclei: septum, accumbens nucleus, and ventrolateral area. Cell labeling varied depending on section location in the telencephalon.

#### 3.5.6 Stages 26–28

The distribution of retrogradely labeled cells in the ipsilateral basal nuclei (Figure 15k–o) after an injection into the forebrain bundles (Figure 15b–g) was shown. Labeled cells were identified in the septum, the accumbens nucleus, and the ventrolateral area. The distribution of labeled neurons in these respective areas depended on the location in the telencephalon. Labeled cells were present as far anteriorly as the rostral pole of the telencephalon (Figure 15o).

## 4 Discussion

Similar to any other tract tracing study, whether performed in embryos or adults, the resulting observations are influenced by the injection site, tracer employed, and survival times. The present study is no different, and the potential impacts of these factors are acknowledged.

The tracer applications mainly involved the forebrain bundles with varying degrees of extension into the diencephalon itself. This had the advantage of labeling exiting axons from multiple nuclei as opposed to tracer applications that were limited to restricted nuclei. A similar strategy was used to investigate afferents to the basal nuclei in juvenile crocodiles. This approach labeled more axons and retrogradely labeled cells than did dorsal thalamic injections themselves (Pritz 2024).

Survival times in the ex vivo setting varied. Before experiments were undertaken, optimal survival times were not known. Accordingly, a range was selected between 1 and 4 days. Brain tissue began to disintegrate after about 4 days. Variations in survival did not seem to influence the results.

### 4.1 Brown Reaction Product

An additional potential concern in interpreting these data was the presence of a brown reaction product of varying size located at a distance from the injection site. Its etiology and its effect on the present results are unclear. The appearance of this brown reaction product differed from the staining that commonly surrounded tracer injections seen in juvenile crocodilians. This brown reaction product could have influenced the present observations. For example, in Figure 6k, the brown reaction product covered nucleus dorsolateralis anterior and might have contributed to labeling of medial forebrain bundle fibers. If so, then labeled axons exiting nucleus dorsolateralis anterior to enter the medial forebrain bundle should have been observed. However, this was not seen. Furthermore, labeled fibers leaving other diencephalic areas covered by this brown reaction product were not found in any of the other cases detailed in this report. While anterograde fibers may not have been labeled, retrogradely labeled cells could have taken up this tracer. However, the location and distribution of basal nuclei projecting cells observed in pre‐hatchlings at Stages 26–28 were similar to those retrogradely labeled cells observed after forebrain bundle injections in juvenile *Caiman crocodilus* (Pritz 2024). The presence of the brown reaction product did not seem to influence the results of structures labeled by the injection.

### 4.2 Synaptic Vesicle 2 (SV2) Immunostaining

At Stage 20, SV2 immunostaining was seen in the optic tectum and hypothalamus but not in the basal nuclei or rostral dorsolateral area until Stage 22. The appearance of these puncta resembled axon terminals observed after tracer injections of horseradish peroxidase (Pritz and Stritzel 1994) or after axonal degeneration after brain lesions in a related crocodilian, *Caiman crocodilus* (Pritz 1974, 1975). Furthermore, the appearance of these puncta was similar to that seen surrounding rat cortex soma after similar processing of tissue as used in these experiments in *Alligator*. Considerable data (summarized in Nealen 2005) support the finding that SV2 immunostaining, using the same mouse‐derived monoclonal antibody employed in the present experiments, represents axon terminals. Together, these data indicate that the puncta observed in telencephalon and elsewhere represent boutons.

### 4.3 Summary of Results

The development of the tracts that interconnect the thalamus with the telencephalon is summarized (Figure 16). These four circuits are: (1) the ipsilateral lateral forebrain bundle, (2) the ipsilateral, (3) contralateral medial forebrain bundle, and (4) the circuit from the basal nuclei to the dorsal thalamus. With the exception of the septum, the telencephalon undergoes differentiation of some structures beginning at Stage 19.5, whereas divisions in the dorsal thalamus are noted at Stage 21.5. While Stage 19.5 might have been selected as a starting point for study, the present observations were made before this time. This strategy was undertaken to determine whether any prior events in development may have influenced the formation of any of these four circuits. Their development is discussed in the order of their occurrence.

**FIGURE 16:**
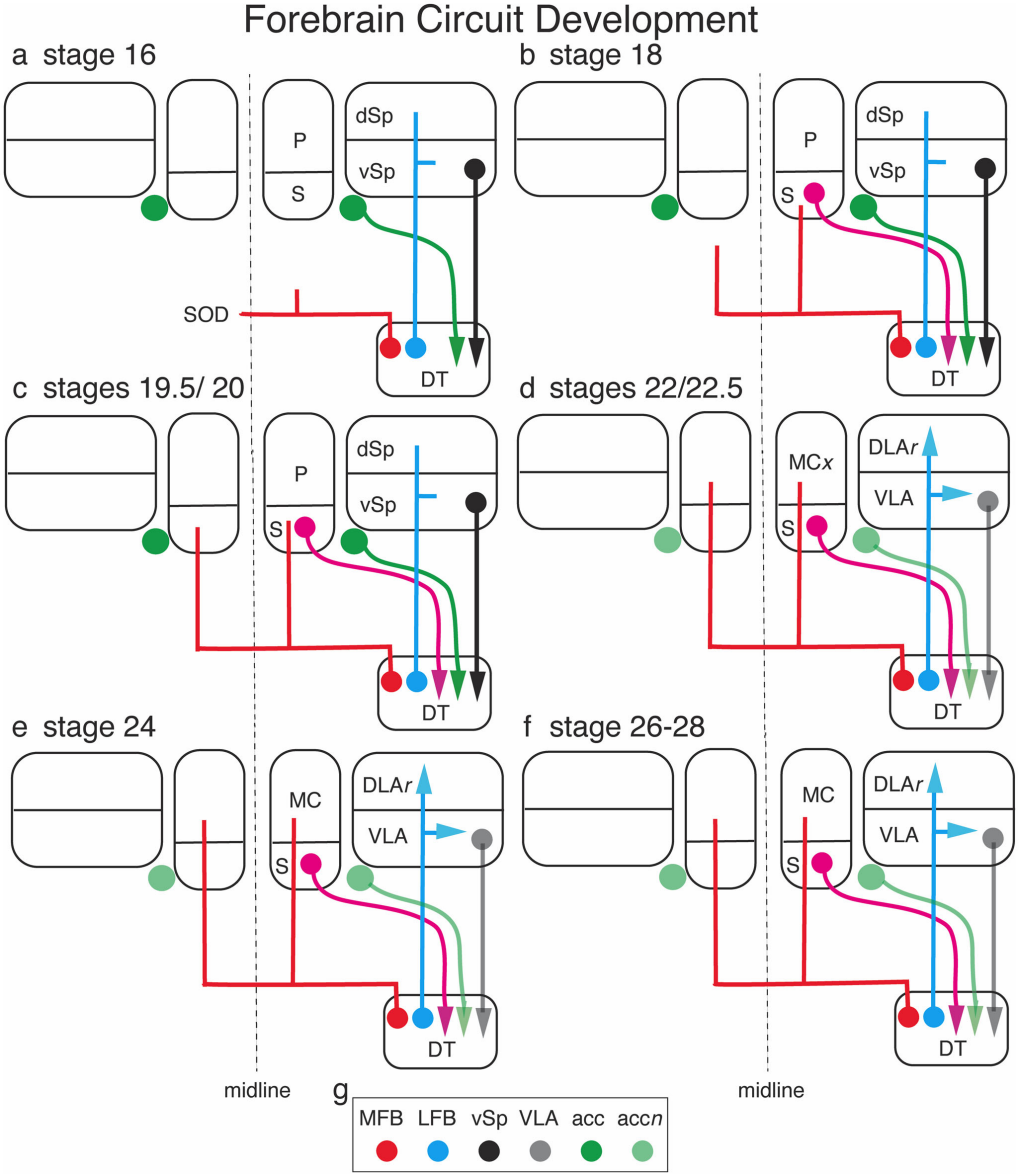
Summary of forebrain circuit development. Schematic diagram, not drawn to scale, illustrates the development of the circuits interconnecting the dorsal thalamus and the telencephalon beginning with Stage 16 (a) and continuing through Stages 26–28 (f). Arrows represent synaptic connections. Color‐coding scheme is shown (g). acc, accumbens area; acc*n*, accumbens nucleus; DLA*r*, rostral dorsolateral area; dSp, dorsal subpallium; DT, dorsal thalamus; LFB, lateral forebrain bundle; MC, medial cortex; MC*x*, medial cortex anlage; MFB, medial forebrain bundle; P, pallium; S, septum; SOD, supraoptic decussation; VLA, ventrolateral area; vSp, ventral subpallium.

### 4.4 Basal Nuclei to the Dorsal Thalamus Circuit

Unexpectedly, the tract from the ventral subpallium, septum, and accumbens area to the diencephalon was the earliest circuit to form. Axons either in the forebrain bundles themselves or in the diencephalon itself must have taken up the tracer in order for cells to be retrogradely labeled in these anlagen of the basal nuclei. This occurred as early as Stage 16 when the ventral subpallium and the accumbens area were present and before they became differentiated.

### 4.5 Lateral Forebrain Circuit

At the earliest developmental time examined, Stage 16, fibers in the lateral forebrain pass through the ventral subpallium and reach the dorsal subpallium. However, it was not until Stage 22 that synapse formation occurred. This time was shortly after differentiation of the dorsal thalamus and telencephalon. Furthermore, at Stage 22, puncta were present and were even organized as terminal fields. These puncta were considered to represent synaptic boutons. Taken together, these observations suggest that information transmitted via the lateral forebrain bundle is likely functional before hatching.

### 4.6 Medial Forebrain Circuit

Fibers in the medial forebrain bundle project bilaterally to reach the telencephalon. The contralateral component crosses in the supraoptic decussation at Stage 18 where ipsilateral fibers reach the septum. At later stages, Stages 20 and 22, axons course along the medial wall of the hemisphere to reach both the septum and the medial cortex anlage. Still later, at Stages 24 and 26–28, fibers extend further along the medial wall of the hemisphere. However, these axons could not be traced to that portion of the medial cortex that abuts the dorsal cortex. Furthermore, clusters of puncta observed in the rostral dorsolateral area were not seen after labeling of the medial forebrain bundle. More fibers were present ipsilaterally than were seen contralaterally. These observations suggest that the ipsilateral and contralateral medial forebrain bundles do not reach their terminus in the cortex before hatching.

### 4.7 Potential Impact of Timing of Circuit Formation

The timing for the formation of these pathways interconnecting the diencephalon with the telencephalon is unexplained. The following suggest some possibilities.

#### 4.7.1 Basal Nuclei to the Dorsal Thalamus Circuit

The tract from the basal nuclei to the dorsal thalamus is the first of these four circuits to form during forebrain development. One explanation for the earliest occurrence of this circuit is the following. This circuit is suggested to form a scaffold for the future lateral and medial forebrain bundle fibers that project from the dorsal thalamus to the telencephalon. Whether other amniotes follow this pattern in this same or even other reciprocal circuits is not known. However, the cortico‐thalamic tract in mammals forms earlier in development than does the reciprocal thalamo‐cortical circuit (Molnár and Kwan 2024). A further test of this suggestion would be to examine the development of thalamo‐cortical and cortico‐thalamic and dorsal thalamus to basal nuclei and basal nuclei to dorsal thalamus circuits in turtles. While considerable data on turtle forebrain circuit development has been published (Cordery and Molnár 1999; Bielle et al. 2011; Tosa et al. 2015), this specific question has not been addressed.

While cells within the fascicles of these forebrain bundles are present and could also provide guidance for these axons, their numbers were few, and they were not prominent until Stage 22. This time period was considerably later than the initial identification of this tract at Stage 16.

In rodents, cortico‐thalamic fibers arrive at the junction of the pallial‐subpallial boundary within the telencephalon before dorsal thalamic axons do. Subsequent co‐fasciculation between the axons from these two sites has been suggested as a partial explanation for the guidance of each tract to its correct target (summarized by Molnár and Kwan 2024). However, this may be a feature of thalamo‐cortical interconnection development restricted to mammals and, perhaps, to turtles, the only reptiles so far known to have this reciprocal circuit. Since the dorsal ventricular ridge is a structure unique to reptiles (Ulinski 1983), the rules for the development of its interconnections may differ from what has been described for mammals.

#### 4.7.2 Lateral and Medial Forebrain Circuits

The timing of fiber development and the appearance of presumed terminals differed between the lateral and medial forebrain bundles. Fibers in the lateral forebrain bundle reached the telencephalon earlier in development than those from the medial forebrain bundle. In addition, puncta, representing likely terminals, were present in the rostral dorsolateral area, often appearing as localized clusters reminiscent of terminal fields. Fibers in the medial forebrain bundle ultimately reached the more inferior portions of the medial cortex but did not project further distally to the medial cortex that abuts the dorsal cortex. Patches of puncta related to medial forebrain bundle fibers were not found along the medial wall of the hemisphere, either in the septum or in the medial cortex. While technical factors could explain these differences between the lateral and medial forebrain bundles, a more likely explanation is that the medial forebrain bundle has not reached its target before hatching. If so, these differences could be related to the type of information carried by these respective circuits. Physiological data in *Alligator* are lacking. However, anatomical tract tracing studies in *Caiman crocodilus* have identified sensory projections from auditory, visual, and somatosensory nuclei, which utilize the lateral forebrain bundle to terminate in localized parts of the rostral dorsolateral area (Pritz 1974,1975; Pritz and Stritzel 1994). Physiological studies of nuclei that use the medial forebrain are also lacking in crocodiles. Nevertheless, one explanation for the differences between these two circuits is that certain sensory information carried by the lateral forebrain bundle is more critical for survival after hatching than is the information transmitted by the medial forebrain bundle.

### 4.8 Comparison With Other Amniotes

Mammals lack a dorsal ventricular ridge and do not have a bilateral thalamo‐telencephalic circuit that uses the medial forebrain bundle. However, mammals have striatal, amygdaloid, and septal regions that are comparable to reptiles, including crocodiles. These structures in mammals have tracts that are comparable to the basal nuclei circuit described for *Alligator* (Medina et al. 2017). Studies describing either the development of the basal nuclei to dorsal thalamus circuit or the development of the medial forebrain bundle have not been investigated in other sauropsids. Accordingly, the comparisons that follow are limited to reports that investigated the development of the connections between the dorsal thalamus and the telencephalon that use the lateral forebrain bundle.

#### 4.8.1 Comparisons With Other Reptiles

The development of the lateral forebrain bundle and its termination in the telencephalon have been investigated in turtles (Cordery and Molnàr 1999; Bielle et al. 2011; Tosa et al. 2015. The focus of these reports has varied. One has extensively detailed the development of thalamo‐telencephalic interconnections through a number of interrelated experiments to provide a wealth of data (Cordery and Molnàr 1999). The other two have provided further information on guidance molecules that influenced the course of the lateral forebrain bundle (Bielle et al. 2011; Tosa et al. 2015). Each of these reports has documented a trajectory and timing of the lateral forebrain bundle projections to the dorsal ventricular ridge that are similar to the present results in *Alligator*.

#### 4.8.2 Comparisons With Birds

Studies in chick have focused on the rotundo‐entopallial (ectostriatal, visual dorsal ventricular ridge) pathway (Wu et al. 2000; Reyes‐Pinto et al. 2024), while other reports have examined the overall formation of the dorsal thalamus to the dorsal ventricular ridge circuit (Bielle et al. 2011; Tosa et al. 2015). Studies focusing on the rotundo‐entopallial pathway have been of two types. One has placed a retrograde tracer into the dorsal ventricular ridge (Wu et al. 2000), whereas the other inserted tracers into nucleus rotundus and examined the ascending connections to the telencephalon (Reyes‐Pinto et al. 2024). Those that are most relevant to the present analysis document the development of connections from nucleus rotundus in particular (Reyes‐Pinto et al. 2024) or from the dorsal thalamus in general to the telencephalon (Bielle et al. 2011; Tosa et al. 2015). In all cases, even when the overall focus of the experiments investigated guidance molecules influencing circuit formation (Bielle et al. 2011; Tosa et al. 2015), the overall pattern of the thalamo‐telencephalic circuit using the lateral forebrain bundle was similar to the present analysis. In fact, in one report, the sagittal images in chick (Reyes‐Pinto et al. 2024; Figures 8 and 9) were remarkably similar to the photos shown for *Alligator* (Figures 7 and 8).

## 5 Concluding Remarks

The present analysis has outlined the development of four circuits that interconnect the dorsal thalamus and the telencephalon. These are: the ipsilateral lateral forebrain bundle, the ipsilateral and contralateral medial forebrain bundle, and a tract from the basal nuclei to the dorsal thalamus. The earliest of these circuits to form is the one from the basal nuclei to the dorsal thalamus. This fiber bundle is suggested to serve as a scaffold for the later developing lateral forebrain bundle and the last to form, the medial forebrain bundle. The lateral and medial forebrain bundles differ not only in their respective time of occurrence but also in the morphology of their fascicles. These differences are suggested to play a role in behavior after hatching.

## Funding

The authors have nothing to report.

## Conflicts of Interest

The authors declare no conflicts of interest.

## Ethics Statement

The experiments in this report were approved by the animal use committee of Indiana University (Protocol Approval 10516). This protocol followed the guidelines of the National Institutes of Health.

## Data Availability

The data that support the findings of this study are available from the corresponding author upon reasonable request.

## References

[cne70203-bib-0001] Beckstead, R. M. 1979. “An Autoradiographic Examination of Corticocortical and Subcortical Projections of the Mediodorsal‐Projection (Prefrontal) Cortex in the Rat.” Journal of Comparative Neurology 184: 43–62. 10.1002/cne.901840104 762282

[cne70203-bib-0002] Belekhova, M. G. , V. D. Zharskaja , A. S. Khachunts , G. V. Gaidaenko , and N. L. Tumanova . 1985. “Connections of the Mesencephalic, Thalamic and Telencephalic Auditory Centers in Turtles. Some Structural Bases for Audiosomatic Interrelations.” Journal Fur Hirnforschung 26: 127–152. 2410486

[cne70203-bib-0003] Bielle, F. , P. Marcos‐Mondejar , M. Keita , et al. 2011. “Slit2 Activity in the Migration of Guidepost Neurons Shapes Thalamic Projections During Development and Evolution.” Neuron 69: 1085–1098. 10.1016/j.neuron.2011.02.026 21435555

[cne70203-bib-0004] Brauth, S. E. , and C. A. Kitt . 1980. “The Paleostriatal System of *Caiman crocodilus* .” Journal of Comparative Neurology 189: 437–465. 10.1002/cne.901890303 7372857

[cne70203-bib-0005] Carretta, D. , A. Sbriccoli , M. Santarelli , F. Pinto , A. Granato , and D. Minciacchi . 1996. “Crossed Thalamo‐Cortical and Cortico‐Thalamic Projections in Adult Mice.” Neuroscience Letters 204: 69–72. 10.1016/0304-3940(96)12319-3 8929980

[cne70203-bib-0006] Cordery, P. , and Z. Molnár . 1999. “Embryonic Development of Connections in Turtle Pallium.” Journal of Comparative Neurology 413: 26–54. 10464368

[cne70203-bib-0007] Davis, R. E. , and B. W. Agranoff . 1977. “Microimplantation of [3H]Proline on a Single Bead of Ion‐Exchange Resin.” Brain Research 124: 341–346. 10.1016/0006-8993(77)90890-3 843951

[cne70203-bib-0008] Dermon, C. R. , and H. Barbas . 1994. “Contralateral Thalamic Projections Predominantly Reach Transitional Cortices in the Rhesus Monkey.” Journal of Comparative Neurology 344: 508–531. 10.1002/cne.903440403 7523458

[cne70203-bib-0009] Desan, P. H. 1988. “Organization of the Cerebral Cortex in Turtle.” In The Forebrain of Reptiles. Current Concepts of Structure and Function, edited by W. K. Schwerdtfeger and W. Smeets , 1–11. Karger.

[cne70203-bib-0010] Desfilis, E. , E. Font , M. Belekhova , and N. Kenigfest . 2002. “Afferent and Efferent Projections of the Dorsal Anterior Thalamic Nuclei in the Lizard *Podarcis hispanica* (Sauria, Lacertidae).” Brain Research Bulletin 57: 447–450. 10.1016/s0361-9230(01)00727-4 11923008

[cne70203-bib-0011] Dinopoulos, A. 1994. “Reciprocal Connections of the Motor Neocortical Area With the Contralateral Thalamus in the Hedgehog (*Erinaceus europaeus)* Brain.” European Journal of Neuroscience 6: 374–380. 10.1111/j.1460-9568.1994.tb00280.x 8019674

[cne70203-bib-0012] Ferguson, M. W. J. 1985. “Reproductive Biology and Embryology of the Crocodilians.” In Biology of the Reptilia, edited by C. Gans ,329–491. John Wiley & Sons.

[cne70203-bib-0013] Guillery, R. 2017. The Brain as a Tool. A Neuroscientist's Account. Oxford University Press.

[cne70203-bib-0014] Hall, J. A. , R. E. Foster , F. F. Ebner , and W. C. Hall . 1977. “Visual Cortex in a Reptile, the Turtle (*Pseudemys scripta* and *Chrysemys picta*).” Brain Research 130: 197–216. 10.1016/0006-8993(77)90270-0 884523

[cne70203-bib-0015] Hall, W. C. , and F. F. Ebner . 1970. “Thalamotelencephalic Projections in the Turtle (*Pseudemys scripta*).” Journal of Comparative Neurology 140: 101–122. 10.1002/cne.901400107 5459208

[cne70203-bib-0016] Hedges, S. B. 1994. “Molecular Evidence for the Origin of Birds.” Proceedings of the National Academy of Sciences 91: 2621–2624. 10.1073/pnas.91.7.2621 PMC43421 8146164

[cne70203-bib-0017] Heller, S. B. , and P. S. Ulinski . 1987. “Morphology of Geniculocortical Axons in Turtles of the Genera *Pseudemys* and *Chrysemys* .” Anatomy and Embryology (Berl) 175: 505–515. 10.1007/BF00309685 2437831

[cne70203-bib-0018] Jones, E. G. 2007. The Thalamus. 2nd ed. Cambridge University Press.

[cne70203-bib-0019] Karten, H. J. , W. Hodos , W. J. Nauta , and A. M. Revzin . 1973. “Neural Connections of the “Visual Wulst” of the Avian Telencephalon. Experimental Studies in the Pigeon (*Columba livia*) and Owl (*Speotyto cunicularia*).” Journal of Comparative Neurology 150: 253–277. 10.1002/cne.901500303 4721779

[cne70203-bib-0020] Künzle, H. 1995. “Crossed Thalamocortical Connections in the Madagascan Hedgehog Tenrec: Dissimilarities to Erinaceous Hedgehog, Similarities to Mammals With More Differentiated Brains.” Neuroscience Letters 189: 89–92. 10.1016/0304-3940(95)11458-9 7541906

[cne70203-bib-0021] Lohman, A. H. M. , and I. van Woerden‐Verkley . 1978. “Ascending Connections to the Forebrain in the Tegu Lizard.” Journal of Comparative Neurology 182: 555–574. 10.1002/cne.901820309 721969

[cne70203-bib-0022] Martinez‐Garcia, F. , and M. J. Lorente . 1990. “Thalamo‐Cortical Projections in the Lizard *Podarcis hispanica* .” Experimental Brain Research series 19: 93–102.

[cne70203-bib-0023] Mathiasen, M. L. , C. M. Dillingham , L. Kinnavane , A. L. Powell , and J. P. Aggleton . 2017. “Asymmetric Cross‐Hemispheric Connections Link the Rat Anterior Thalamic Nuclei With the Cortex and Hippocampal Formation.” Neuroscience 349: 128–143. 10.1016/j.neuroscience.2017.02.026 PMC5387186 28237814

[cne70203-bib-0024] Medina, L. , A. Abellán , and E. Desfilis . 2021. “Evolving Views on the Pallium.” Brain, Behavior and Evolution 96: 181–199. 10.1159/000519260 34657034

[cne70203-bib-0025] Medina, L. , A. Abellán , A. Vicario , B. Castro‐Robles , and E. Desfilis . 2017. “The Amygdala.” In Evolution of Nervous Systems, 2nd ed., edited by J. Kaas , 427–478. Elsevier.

[cne70203-bib-0026] Molinari, M. , D. Minciacchi , M. Bentivoglio , and G. Macchi . 1985. “Efferent Fibers From the Motor Cortex Terminate Bilaterally in the Thalamus of Rats and Cats.” Experimental Brain Research 57: 305–312. 10.1007/BF00236536 3972032

[cne70203-bib-0027] Molnár, Z. , and K. Y. Kwan . 2024. “Development and Evolution of Thalamocortical Connectivity.” Cold Spring Harbor Perspectives in Biology 16: a041503. 10.1101/cshperspect.a041503 PMC10759993 38167425

[cne70203-bib-0028] Mulligan, K. A. , and P. S. Ulinski . 1990. “Organization of Geniculocortical Projections in Turtles: Isoazimuth Lamellae in the Visual Cortex.” Journal of Comparative Neurology 296: 531–547. 10.1002/cne.902960403 2358551

[cne70203-bib-0029] Nealen, P. M. 2005. “An Interspecific Comparison Using Immunofluorescence Reveals That Synapse Density in the Avian Song System Is Related to Sex but Not to Male Song Repertoire Size.” Brain Research 1032: 50–62. 10.1016/j.brainres.2004.10.059 15680941

[cne70203-bib-0030] Ouimet, C. C. , R. L. Patrick , and F. F. Ebner . 1985. “The Projection of Three Extrathalamic Cell Groups to the Cerebral Cortex of the Turtle Pseudemys.” Journal of Comparative Neurology 237: 77–84. 10.1002/cne.902370106 2995458

[cne70203-bib-0030a] Parent, A. 1976. “Striatal Afferent Connections in the Turtle (*Chrysemys picta*) as revealed by Retrograde Axonal Transport of Horseradish Peroxidase.” Brain Research 108: 25–36. 10.1016/0006-8993(76)90161-x 1276890

[cne70203-bib-0031] Perez‐Santana, L. , O. Marín , and W. J. A. J. Smeets . 1997. “Afferent Connections of the Nucleus Accumbens of the Snake, *Elaphe guttata*, Studied by Means of In Vitro and In Vivo Tracing Techniques in Combination With TH Immunohistochemistry.” Neuroscience Letters 225: 101–104. 10.1016/s0304-3940(97)00205-x 9147384

[cne70203-bib-0032] Preuss, T. M. , and P. S. Goldman‐Rakic . 1987. “Crossed Corticothalamic and Thalamocortical Connections of Macaque Prefrontal Cortex.” Journal of Comparative Neurology 257: 269–281. 10.1002/cne.902570211 3571529

[cne70203-bib-0033] Pritz, M. B. 1974. “Ascending Connections of a Thalamic Auditory Area in a Crocodile, *Caiman Crocodilus* .” Journal of Comparative Neurology 153: 199–213. 10.1002/cne.901530205 4359289

[cne70203-bib-0034] Pritz, M. B. 1975. “Anatomical Identification of a Telencephalic Visual Area in Crocodiles: Ascending Connections of Nucleus Rotundus in *Caiman Crocodilus* .” Journal of Comparative Neurology 164: 323–338. 10.1002/cne.901640305 1184787

[cne70203-bib-0035] Pritz, M. B. 2024. “Interconnections Between the Dorsal Thalamus and the Basal Nuclei in a Reptile.” Neuroscience Letters 836: 137894. 10.1016/j.neulet.2024.137894 38997083

[cne70203-bib-0036] Pritz, M. B. 2025a. “Development of the Dorsal Thalamus in a Reptile: Identification of Subdivisions and Their Associated Nuclei.” International Journal of Developmental Biology 69: 109–122. 10.1387/ijdb.250018mp 40928058

[cne70203-bib-0037] Pritz, M. B. 2025b. “Early Development of a Vertebrate Telencephalon: Observations Before Areal Differentiation.” Brain Mechanisms 145–147: 202509.

[cne70203-bib-0038] Pritz, M. B. 2025c. “Later Development of a Vertebrate Telencephalon: Time of Origin of Identified Subdivisions, Derived Nuclei, and Associated Tracts.” Brain Mechanisms 148–150: 202520.

[cne70203-bib-0039] Pritz, M. B. 2026. “Early Development of the Habenula.” Brain Mechanisms 153: 202544.

[cne70203-bib-0040] Pritz, M. B. , and M. E. Stritzel . 1994. “Anatomical Identification of a Telencephalic Somatosensory Area in a Reptile, *Caiman crocodilus* .” Brain Behavior and Evolution 43: 107–127. 10.1159/000113628 8143143

[cne70203-bib-0041] Regidor, J. , and I. Divac . 1992. “Bilateral Thalamocortical Projection in Hedgehogs: Evolutionary Implications.” Brain Behavior and Evolution 39: 265–269. 10.1159/000114123 1498648

[cne70203-bib-0042] Reiner, A. , and H. J. Karten . 1983. “The Laminar Source of Efferent Projections From the Avian Wulst.” Brain Research 275: 349–354. 10.1016/0006-8993(83)90996-4 6194858

[cne70203-bib-0043] Reyes‐Pinto, R. , M.‐J. Rojas , J.‐C. Letelier , G. J. Marín , and J. Mpodozis . 2024. “Early Development of the Thalamo‐Pallial Stage of the Tectofugal Visual Pathway in the Chicken (*Gallus gallus*).” Journal of Comparative Neurology 532: e25657. 10.1002/cne.25657 38987912

[cne70203-bib-0044] Romero‐Alemán, M. M. , M. Monzón‐Mayor , E. Santos , and C. Yanes . 2010. “Expression of Neuronal Markers, Synaptic Proteins, and Glutamine Synthetase in the Control and Regenerating Lizard Visual System.” Journal of Comparative Neurology 518: 4067–4087. 10.1002/cne.22444 20737600

[cne70203-bib-0045] Sherman, S. M. , and R. W. Guillery . 2013. Functional Connections of Cortical Areas. A New View From the Thalamus. MIT Press.

[cne70203-bib-0046] Shoer Goldman, P. 1979. “Contralateral Projections to the Dorsal Thalamus From Frontal Association Cortex in the rhesus Monkey.” Brain Research 166: 166–171. 10.1016/0006-8993(79)90658-9 105784

[cne70203-bib-0047] Siemen, M. , and H. Künzle . 1994. “Connections of the Basal Telencephalic Areas C and D in the Turtle Brain.” Anatomy and Embryology 189: 339–359. 10.1007/BF00190590 8074323

[cne70203-bib-0048] Subhedar, N. , N. S. R. Krishna , and P. D. P. Rao . 1989. “Cytoarchitectonic Pattern of the Hypothalamus in the Crocodile, *Gavialis gangeticus* .” Cell And Tissue Research 255: 89–105.

[cne70203-bib-0049] Tosa, Y. , A. Hirao , I. Matsubara , et al. 2015. “Development of the Thalamo‐Dorsal Ventricular Ridge Tract in the Chinese Soft‐Shelled Turtle, *Pelodiscus sinensis* .” Development, Growth & Differentiation 57: 40–57. 10.1111/dgd.12186 25494924

[cne70203-bib-0050] Ulinski, P. S. 1983. Dorsal Ventricular Ridge. A Treatise on Forebrain Organization in Reptiles and Birds. John Wiley & Sons.

[cne70203-bib-0051] Ulinski, P. S. 1986. “Organization of Corticogeniculate Projections in the Turtle, *Pseudemys scripta* .” Journal of Comparative Neurology 254: 529–542. 10.1002/cne.902540406 3805361

[cne70203-bib-0052] Whetstone, K. N. , and L. D. Martin . 1979. “New Look at the Origin of Birds and Crocodiles.” Nature 279: 234–236. 10.1038/279234a0 440432

[cne70203-bib-0053] Wu, C. C. , R. K. Charlton , and H. J. Karten . 2000. “The Timecourse of Neuronal Connections of the Rotundoectostriatal Pathway in Chicks (*Gallus gallus*) During Embryogenesis: A Retrograde Transport Study.” Visual Neuroscience 17: 905–909. 10.1017/s0952523800176096 11193106

[cne70203-bib-0054] Wu, C. C. , and H. J. Karten . 1998. “The Thalamo‐Hyperstriatal System Is Established by the Time of Hatching in Chicks (*Gallus gallus*): A Cholera Toxin B Subunit Study.” Visual Neuroscience 15: 349–358. 10.1017/s0952523898152148 9605534

[cne70203-bib-0055] Zhu, D. , K. H. Lustig , K. Bifulco , and J. Keifer . 2005. “Thalamocortical Connections in the Pond Turtle *Pseudemys scripta elegans* .” Brain, Behavior and Evolution 65: 278–292. 10.1159/000084317 15761219

